# Flow and hydrodynamic shear stress inside a printing needle during biofabrication

**DOI:** 10.1371/journal.pone.0236371

**Published:** 2020-07-24

**Authors:** Sebastian J. Müller, Elham Mirzahossein, Emil N. Iftekhar, Christian Bächer, Stefan Schrüfer, Dirk W. Schubert, Ben Fabry, Stephan Gekle

**Affiliations:** 1 Biofluid Simulation and Modeling, Universität Bayreuth, Bayreuth, Germany; 2 Department of Phyiscs, Friedrich-Alexander Universität Erlangen-Nürnberg, Erlangen, Germany; 3 Institute of Polymer Materials, Friedrich-Alexander Universität Erlangen-Nürnberg, Erlangen, Germany; 4 KeyLab Advanced Fiber Technology, Bavarian Polymer Institute, Fürth, Germany; University of New South Wales, AUSTRALIA

## Abstract

We present a simple but accurate algorithm to calculate the flow and shear rate profile of shear thinning fluids, as typically used in biofabrication applications, with an arbitrary viscosity-shear rate relationship in a cylindrical nozzle. By interpolating the viscosity with a set of power-law functions, we obtain a mathematically exact piecewise solution to the incompressible Navier-Stokes equation. The algorithm is validated with known solutions for a simplified Carreau-Yasuda fluid, full numerical simulations for a realistic chitosan hydrogel as well as experimental velocity profiles of alginate and chitosan solutions in a microfluidic channel. We implement the algorithm in an easy-to-use Python tool, included as Supplementary Material, to calculate the velocity and shear rate profile during the printing process, depending on the shear thinning behavior of the bioink and printing parameters such as pressure and nozzle size. We confirm that the shear stress varies in an exactly linear fashion, starting from zero at the nozzle center to the maximum shear stress at the wall, independent of the shear thinning properties of the bioink. Finally, we demonstrate how our method can be inverted to obtain rheological bioink parameters *in-situ* directly before or even during printing from experimentally measured flow rate versus pressure data.

## Introduction

Biofabrication, or bioprinting, is a novel technology aimed at applying common 3D printing techniques to fabricate living tissues. In extrusion-based biofabrication, the survival and functionality of printed cells strongly depend on the hydrodynamic stresses that the cells experience during printing [1–5]. These stresses arise mainly from viscous shear forces in the printer nozzle and are thus directly related to the flow profile and the viscosity of the bioink [6–11] in which the cells are suspended. In an effort to reduce hydrodynamics stresses, shear thinning bioinks have been designed that exhibit a nearly flat velocity profile and correspondingly low shear rates in the nozzle center, in contrast to purely Newtonian liquids that develop a parabolic flow profile with higher shear rates throughout most of the nozzle [12–19]. Consequently, cells suspended in shear thinning bioinks can be expected to show increased survival rate and better functionality after printing [4, 16, 20, 21].

To describe the rheology of inelastic, time-independent, shear thinning materials, a variety of viscosity models exists, which are collectively labeled as generalized Newtonian fluids [22]. One of the simplest models assumes a power-law, also known as Ostwald-de Waele relationship [4, 23, 24]. Real shear thinning materials, however, show power-law behavior only in a limited range of shear rates, while Newtonian behavior is observed above and below this range. The latter is particularly relevant for bioprinting applications and prevails in the central region of the printing nozzle where the velocity approaches a constant value and thus a vanishing shear rate. To properly model this behavior, a widely used description is the Carreau-Yasuda (CY) [22, 25] model, which features a central power-law region that smoothly transitions into two Newtonian plateaus in the limits of low and high shear rates. Many commonly used hydrogel materials for bioprinting [26] but also polymer melts or solutions [27] can be accurately characterized with the CY model. Existing methods to calculate theoretically the velocity profile in the printing nozzle for a CY fluid [28, 29] require the shear rate at the nozzle wall as an input parameter. Experimentally, however, this quantity is usually not known. Instead either the pressure difference or the volume flux serve as control parameter.

In this work, we present an algorithm to compute the full velocity, shear rate, and viscosity profile in a printing nozzle for generalized Newtonian fluids such as shear thinning bioinks. Our algorithm is based on interpolating an arbitrary viscosity-shear rate relation by piecewise continuous power-law functions, and requires only the experimentally imposed printing parameters such as the channel radius and the driving pressure difference or flow rate as input values. To allow for an efficient application of our method in everyday laboratory work, we provide a user-friendly implementation of our algorithm for CY fluids as a Python tool included as S1 File. This tool is much simpler to use than typical computational fluid dynamics software and at the same time can provide higher accuracy at much less computational load. The calculated shear stresses are a measure for the mechanical load experienced by cells embedded in the bioink and can thus directly be correlated to post-printing cell viability measurements [1, 11]. We confirm that the well-known linear shear stress distribution found in Newtonian pipe flow is also valid for shear thinning fluids. We validate our algorithm by comparing it to the exact solution for a simplified Carreau-Yasuda fluid, to full numerical Lattice Boltzmann simulations for a realistic chitosan hydrogel under typical printing conditions, and to experimental velocity profiles of a shear thinning alginate solution in a microfluidic channel. Furthermore, we show how our method can be inverted to construct a capillary rheometer, which allows users to determine the rheological parameters of a given bioink using only a bioprinter and a standard laboratory scale without the need of a sophisticated rheometer. Such *in-situ* measurements of bioink rheology combined with the calculation of expected shear rates will help users to optimize the printing process and to achieve the desired printing results especially when bioprinting shear stress-sensitive living cells.

## 1 Theory and results

### 1.1 Viscosity model

**Fig 1:**
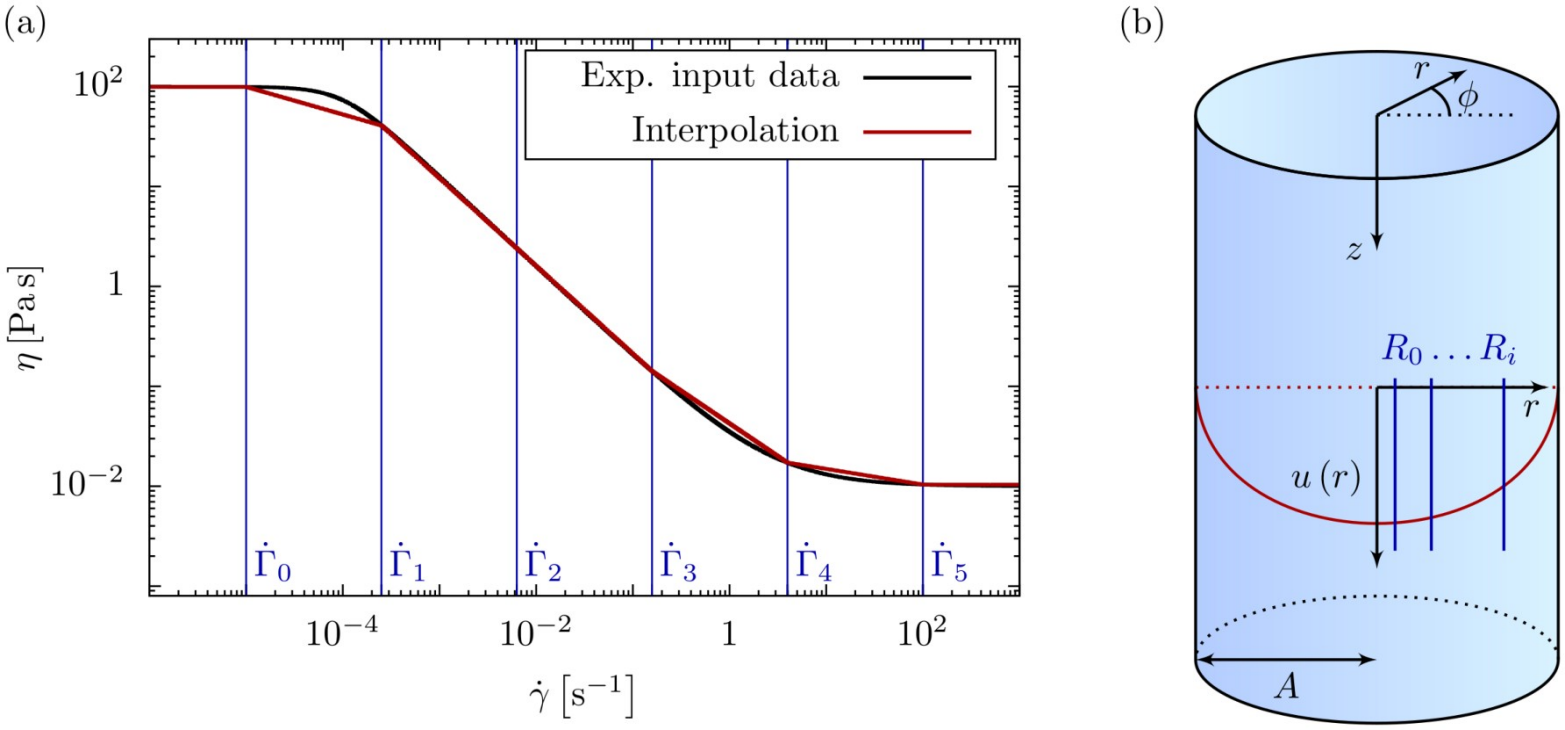
Viscosity and flow profile interpolation. (a) The viscosity-shear rate relationship of an arbitrary shear thinning fluid obtained e. g. from a rheometer measurement is interpolated by power-law intervals. The bounds of the intervals (vertical lines) are given by the intermediate shear rates, ${\dot{\Gamma }}_{i}$. By using a large number of intervals, any arbitrary viscosity-shear rate relationship can be approximated as closely as desired. (b) A long cylinder with uniaxial, stationary flow is used as a model for the flow of a bioink through a printer nozzle. The flow profile is split into radial intervals *R*_*i*_ determined implicitly via the intermediate shear rates ${\dot{\Gamma }}_{i}$.

Our algorithm starts from an experimentally known viscosity-shear rate relation $\eta (\dot{\gamma })$ and interpolates it by a series of power-law functions. The viscosity-shear rate relationship of the bioink, e. g. a cell-laden hydrogel, or any other generalized Newtonian fluid, can be approximated by a continuous, piecewise function as given in (S-1) and depicted in Fig 1a. In every interval, the viscosity-shear rate relation is described by a power-law model $\eta_{i}(\dot{\gamma })=K_{i}{\dot{\gamma }}^{n_{i}-1}$ with a consistency parameter *K*_*i*_ and a dimensionless exponent *n*_*i*_. The *i*^th^ interval is bounded by the shear rates ${\dot{\Gamma }}_{i-1}$ and ${\dot{\Gamma }}_{i}$, and we demand $\eta (\dot{\gamma })$ to be continuous at these bounds.

This continuity condition together with a set of ${\dot{\Gamma }}_{i}$ uniquely determines the power-law parameters *K*_*i*_ and *n*_*i*_ in every interpolation interval, as detailed in section S-1.2.

We note that this approach can be applied to any material described in terms of generalized Newtonian fluids, including yield stress fluids. Furthermore, our method includes cell-laden bioinks, as, on the one hand, the presence of cells has been shown to only slightly alter the materials’ rheological behavior [5, 30]. On the other hand, the macroscopic rheology of a cell suspension, determined e. g. via shear rheometry or our capillary rheometry method presented in section 3, can be used as input for our method.

### 1.2 Governing equations

Analogously to the well-known Poiseuille flow of a Newtonian fluid [31, pp. 180 ff.], we assume a stationary, laminar, and pressure driven flow, with the velocity having only an axial component *u* depending on the radial position *r*. We consider a cylindrical channel and neglect entrance and exit effects. Applying these flow conditions, the incompressible Navier-Stokes equations reduce to the ordinary differential equation as shown in section S-1.3:

$$\begin{array}{c} G=\frac{1}{r}\frac{\partial }{\partial r}(r\eta (\dot{\gamma })\frac{\partial u}{\partial r}) \end{array}$$ (1)

Here, the constant pressure gradient $G≔\frac{\partial p}{\partial z}=\frac{\Delta p}{L}$ is defined by the pressure drop Δ*p* = *p*_0_ − *p*_*L*_ < 0 over a channel segment of length *L*. For a Newtonian fluid, i. e. $\eta (\dot{\gamma })=\eta$, integration of Eq (1) directly yields the well-known linear radial dependency of the shear stress:

$$\begin{array}{c} \sigma (r)=\eta \dot{\gamma }=-\frac{G}{2}r \end{array}$$ (2)

Similar to the piecewise viscosity model in Fig 1a, we decompose the axial velocity *u*(*r*) and the shear rate $\dot{\gamma }(r)$ into radial intervals *R*_*i*_ as given in (S-29) and (S-32) and illustrated in Fig 1b for the velocity.

Inserting these piecewise profiles into the Navier-Stokes equation Eq (1) yields the following system of equations where *i* denotes the intervals as above:

$$G=\frac{1}{r}\frac{\partial }{\partial r}[-rK_{i}({\dot{\gamma }}_{i}(r){)}^{n_{i}}]$$ (3)

$${\dot{\gamma }}_{i}(r)=-\frac{\partial u_{i}(r)}{\partial r}$$ (4)

In order to solve this system of equations we assume the axial velocity to be continuously differentiable and the shear rate to be continuous. The flow shall further fulfill a no-slip boundary condition at the channel wall and have its maximum at the channel center. The mathematical solution to the system of equations Eqs (3) and (4) is detailed in section S-1.4 and section S-1.5 and can be summarized as follows: the shear rate profile is obtained by integrating Eq (3) over the radial position once. Inserting this solution into Eq (4) yields the velocity profile after another integration over *r*. Both integrations come along with integration constants that are determined employing the boundary conditions of the flow and the continuity conditions as stated above.

### 1.3 Results

The first equation Eq (3) can be rearranged and integrated once to obtain the shear rate profile in the *i*^th^ interval:

$$\begin{array}{c} {\dot{\gamma }}_{i}(r)=(-\frac{G}{2K_{i}}r{)}^{\frac{1}{n_{i}}} \end{array}$$ (5)

From this, the velocity profile is obtained by integrating over *r*, which ultimately yields (cf. (S-58)):

$$\begin{array}{cc} u_{i}(r)= & -(-\frac{G}{2K_{i}}{)}^{\frac{1}{n_{i}}}\frac{n_{i}}{n_{i}+1}r^{1+\frac{1}{n_{i}}}\\ & +(-\frac{G}{2K_{k}}{)}^{\frac{1}{n_{k}}}\frac{n_{k}}{n_{k}+1}A^{1+\frac{1}{n_{k}}}\\ & -\sum_{j=i}^{k-1}R_{j}{\dot{\Gamma }}_{j}(\frac{n_{j+1}}{n_{j+1}+1}-\frac{n_{j}}{n_{j}+1}) \end{array}$$ (6)

Here, the newly introduced index *k* denotes the radial interval that contains the physical boundary of the channel, i. e. *R*_*k*−1_ ≤ *A* ≤ *R*_*k*_ with the channel radius *A*.

The radial shear stress profile can, similarly to the Newtonian case, be derived from Eq (1), yielding the same linear behavior:

$$\begin{array}{c} \sigma (r)=-\eta (\dot{\gamma })\frac{\partial u}{\partial r}=-\frac{G}{2}r \end{array}$$ (7)

This shows that the shear stress profile in a cylindrical channel is independent of the shear thinning properties of the material.

Using the solutions for the shear rate (5) and the velocity (6), we derive mathematical expressions for the flow rate as well as the average velocity, shear rate, viscosity, and shear stress. Details of the derivation and the corresponding solutions can be found in (S-63), (S-66), (S-71) and (S-74), respectively (cf. section S-1.6). The flow rate or, equivalently, the average flow velocity determines the printing speed in 3D bioprinting processes. The average shear rate and shear stress can be used to estimate cell damage during printing [2, 4] as detailed in section S-1.8 of the S1 File. We discuss the inclusion of possible wall-slip effects [35] in section S-1.9 of the S1 File.

## 2 Validation

To validate our method, we implement the presented algorithm in a Python [32] tool, included as S1 File together with an explanatory tutorial in section S-3 and available at https://github.com/sjmuellerbt/CYprofiles. Our tool performs the viscosity interpolation according to section 1.1 for a five-parameter Carreau-Yasuda fluid, given in Eq (9). The radial profiles for velocity, shear rate, viscosity, and shear stress and their respective averaged quantities are calculated after providing the printing parameters, i. e. the nozzle radius and the pressure gradient or an imposed flow rate.

We first validate our algorithm using an exact global mathematical solution for a simplified CY model. Next, we compare our algorithm with Lattice Boltzmann simulations for a general CY model using the open source software package ESPResSo [33, 34], for which we extended both the CPU and GPU implementation with several inelastic viscosity models, including the CY model. We finally perform experimental velocity profile measurements in a microfluidic channel and confirm the theoretical prediction of our Lattice Boltzmann simulations.

### 2.1 Validation with global solution

**Fig 2:**
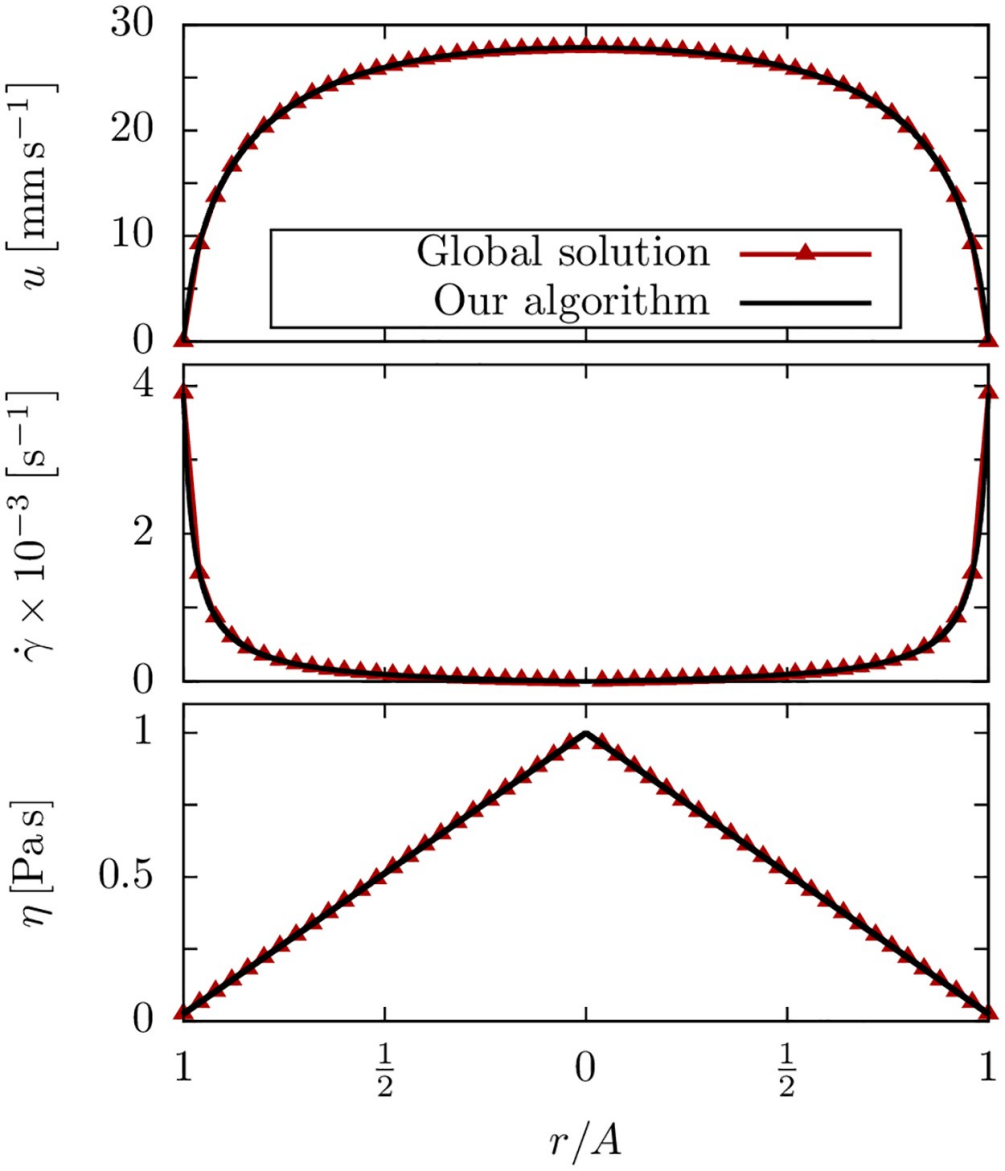
Validation with a mathematical solution. Flow profiles for the simplified CY model: the global mathematical solution and the prediction by our algorithm agree very well. The parameters are *N* = 1000, *η*_0_ = 100 Pa s, *K* = 1.0 s and *G* = −1.95 × 10^6^ Pa m^−1^.

We consider a simplified Carreau-Yassuda (CY) model of the following form

$$\begin{array}{c} \tilde{\eta }(\dot{\gamma })=\frac{{\tilde{\eta }}_{0}}{1+K\dot{\gamma }} \end{array}$$ (8)

where ${\tilde{\eta }}_{0}$ is the viscosity in the limit of zero shear rate and *K* is a time constant. For this model, an exact global solution to the NSE Eq (1) can be found as described in(S-79) and (S-81) (cf. section S-1.7). As shown in Fig 2, we find excellent agreement between this exact solution and the calculated profiles using our Python tool.

### 2.2 Validation with Lattice Boltzmann simulations

The general CY model [25] is given by

$$\begin{array}{c} \tilde{\eta }\left(\dot{\gamma }\right)={\tilde{\eta }}_{\infty }+\frac{{\tilde{\eta }}_{0}-{\tilde{\eta }}_{\infty }}{{\left[1+{\left(K\dot{\gamma }\right)}^{a_{1}}\right]}^{\frac{a_{2}}{a_{1}}}}\;, \end{array}$$ (9)

where ${\tilde{\eta }}_{\infty }$ is the viscosity in the limit of infinite shear rates and the exponents *a*_1_ and *a*_2_ determine the shape of the transition between the zero-shear Newtonian plateau and the power-law region as well as the power-law behavior. For this general CY fluid a global mathematical solution to the NSE does not exist. We therefore compare our algorithm to Lattice-Boltzmann simulations using realistic bioink and printing parameters for a chitosan hydrogel taken from [26] with the following rheological parameters: ${\tilde{\eta }}_{0}=5807\;\text{Pas}$, *K* = 5.33 s, *a*_1_ = 1.35 and *a*_2_ = 0.87. The simulation setup consists of a 5 × 400 × 400 (*x* × *y* × *z*) box with a cylindrical boundary along the *x*-axis corresponding to a physical radius of *A* = 100 μm. The flow is periodic in *x*-direction thus leading to an effectively infinitely long channel. Further details of the Lattice-Boltzmann simulations are given in the S1 File.

**Fig 3:**
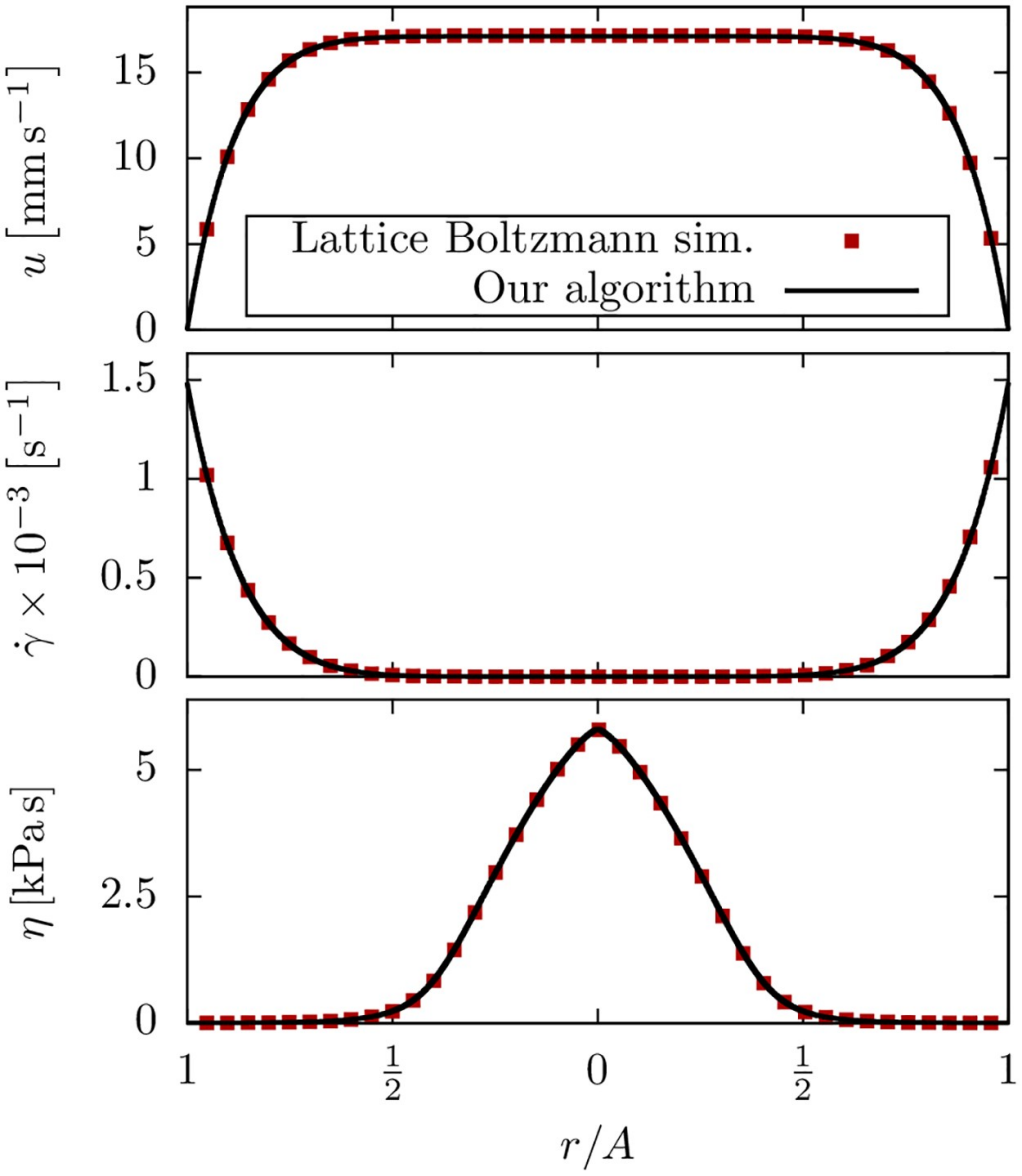
Validation with Lattice Boltzmann calculations. Flow profiles of a chitosan hydrogel with a pressure gradient of *G* = −7.0 × 10^7^ Pa m^−1^ and *N* = 1000.

The calculated and simulated flow profiles are in excellent agreement (Fig 3) thus validating our algorithm for a general CY fluid.

### 2.3 Validation with experimental flow profile measurements

As experimental proof, we measure the flow profile of an alginate solution along the centerline of a microchannel and compare our findings to Lattice Boltzmann simulations of the same geometry.

We prepare a 2.0% alginate solution by mixing 800 mg of alginate (Grindsted PH 176, Dupont, USA) in 50 ml Dulbecco’s phosphate buffered saline under constant stirring overnight at room temperature together with yellow-green fluorescent beads (FluoroSphere carboxylated beads, Invitrogen, diameter: 0.5 μm). The alginate solution is injected under defined pressure into a polymethylmetacrylate microfluidic channel equipped with male mini Luer lock connectors (Darwin Microfluidics, France, internal volume: 8 μl) via a 15 cm long silicon tube (inner diameter: 1 mm). The channel has a length of 58 mm and a quadratic cross section of 190 μm × 190 μm, similar in size to the cross section of a typical printing needle. A square cross section of the channel was chosen to avoid optical distortions that would arise from the curvature of a cylindrical glass capillary in combination with the refractive index differences between glass and alginate. We visualize the flow of alginate using an epifluorescence microscope (DM4, Leica Microsystems, Germany) equipped with a CCD camera (frame rate: 100 Hz, Prosilica GE680, Allied Vision, Germany) and a 100 mW laser diode (473 nm). The microscope is focussed at the mid-section of the channel (height: 95 μm).

**Fig 4:**
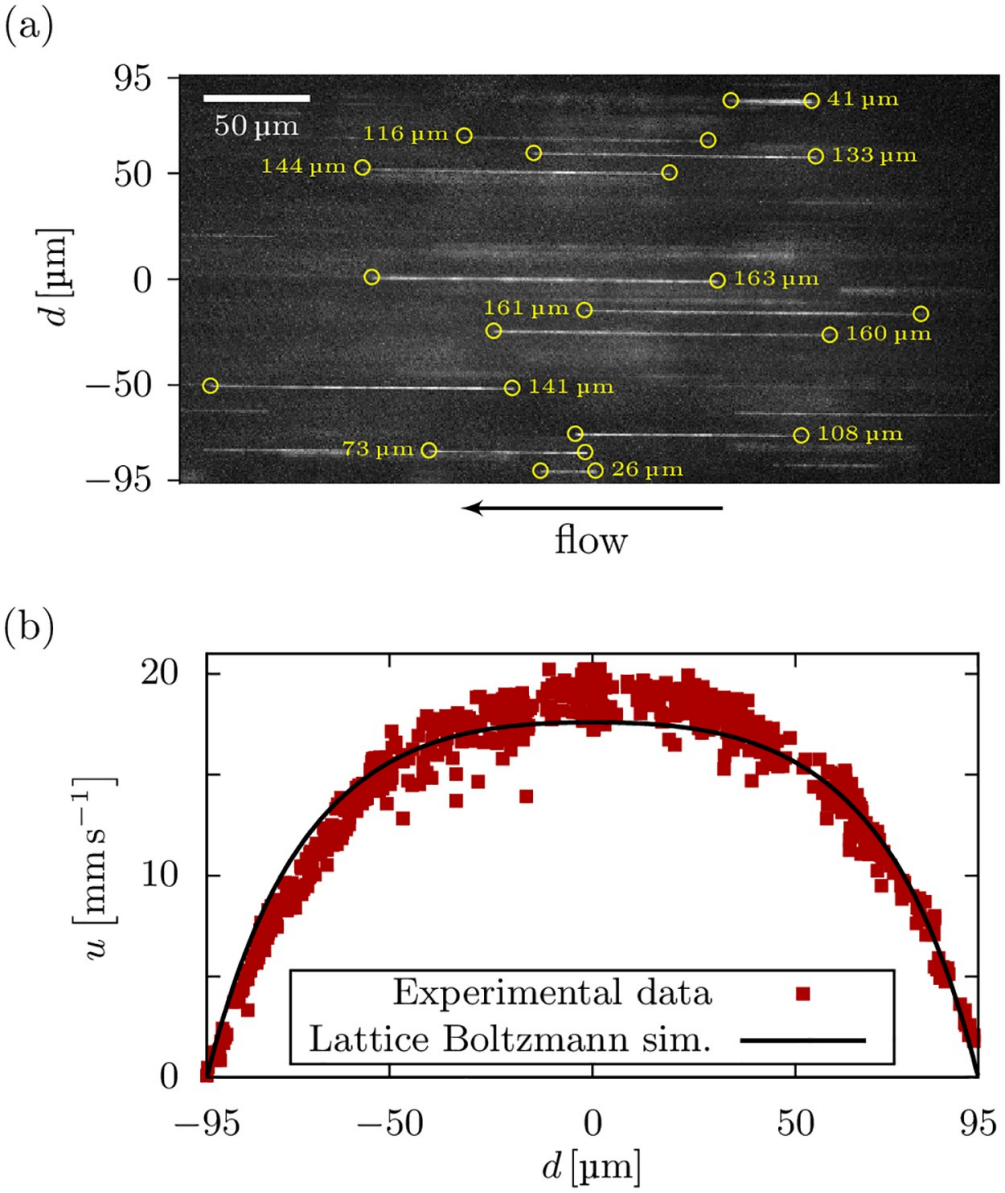
Validation with experimental flow measurements. Experimental measurement of the flow profile of a 2% alginate solution in a 190 μm × 190 μm microchannel. (a) Example micrograph of the bead tracking procedure. The velocity with respect to the lateral position is obtained as the length (yellow circles and labels) of the streaks divided by the exposure time. (b) The measured flow profile is in excellent agreement with our Lattice Boltzmann simulations.

We perform measurements at a pressure of 300 kPa, close to actual printing conditions. The maximum flow speed in the center of the channel is around 2 cm s^−1^, which is too fast to track the beads between successive frames. Instead, the velocity is estimated from the length of the linear streaks of the beads during exposure, as shown in Fig 4a, divided by the exposure time of 7 ms.

We perform Lattice Boltzmann simulations of the pressure driven flow of the alginate solution in a square microchannel. The simulation setup consists of a 5 × 400 × 400 (*x* × *y* × *z*) box with plane boundaries in *y*- and *z*-direction forming a square channel that corresponds to the 190 μm × 190 μm microfluidic channel used in the experiment. The viscosity parameters were obtained using our capillary rheometry method described in section 3 as ${\tilde{\eta }}_{0}=3.65\;\text{Pas}$, ${\dot{\gamma }}_{c}=21.71\;{\text{s}}^{-1}$ and *α* = 0.67, according to equation Eq (10) below. Since we do not know the pressure drop across the mini Luer-lock connectors and tubings, we estimate the pressure gradient from the maximum flow speed measured at the center of the channel. Accordingly, we find that 79% of the total pressure drop of 300 kPa occurs across the 58 mm long channel, while the mini Luer-lock connectors and tubings account for the remaining 21%.

Fig 4b depicts the measured flow profile in comparison to our Lattice Boltzmann simulations. We see excellent agreement of the measured velocity profile and our numerical prediction. Further measurements of alginate hydrogels with different concentrations, as well as a chitosan hydrogel, at various printing pressures and in different channel geometries are included as S1 File.

## 3 Inverse application for a capillary rheometer

Not all laboratories working in bioprinting may have access to sophisticated rheometers for measuring the non-linear viscosity of their bioinks. Moreover, bioinks are often highly sensitive fluids with a large batch-to-batch variation, and the sample used for rheometer measurements may not behave in the same way as the sample used for the actual printing process. In this section, we show how our method can be inverted to perform *in-situ* capillary rheometry measurements using only a bioprinter and a standard laboratory scale. For this, we measure the pressure versus flow rate relationship [35] for a range of discrete pressure values. Using our Python tool, we then extract from this data the non-linear viscosity parameters of the bioink.

### 3.1 Experimental setup

**Fig 5:**
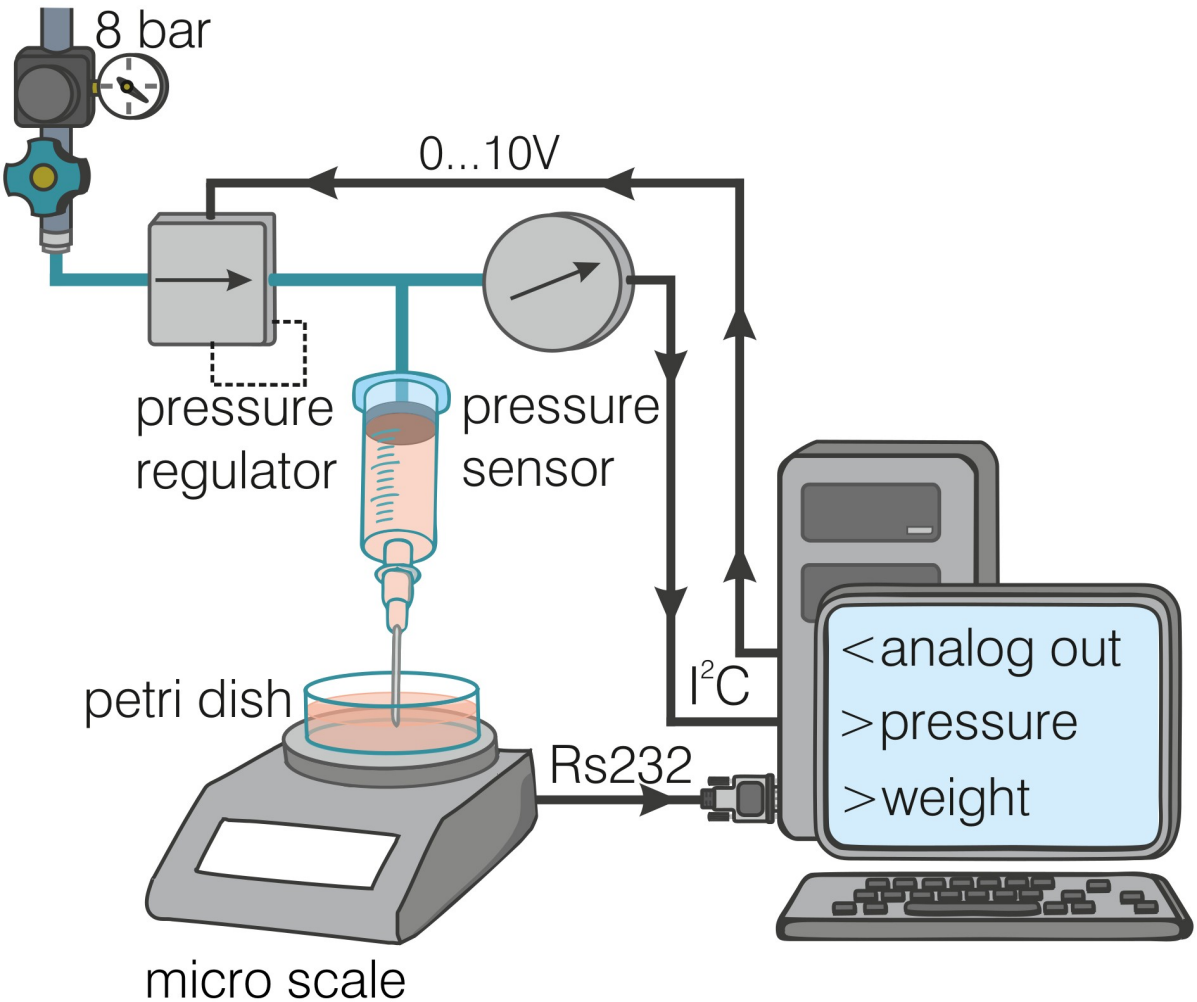
Experimental capillary rheometer setup. Schematic of the experimental setup using a custom-made bioprinter as capillary rheometer: the bioink is driven through a syringe under defined pressure, and the flow rate of the extruded alginate is measured with a precision scale.

We prepare a 2.5% alginate solution using the protocol described in section 2.3 without the addition of fluorescent beads. We measure the viscosity of the alginate solution at a temperature of 25°C at shear rates between 0.01 s^−1^ and 100 s^−1^ using a cone-plate rheometer (DHR-3, TA-Instruments, USA). Alternatively, we measure the viscosity with a custom-made bioprinter that we use here as a capillary rheometer. A schematic of the experimental setup is shown in Fig 5. The alginate solution is driven with a defined pressure (K8P electronic pressure regulator, Camozzi Automation, Italy) through a steel needle (21G blunt cannula #9180109-02, B-Braun, Germany, 28 mm length, 551 μm inner diameter). The pressure is increased stepwise from 20 kPa to 200 kPa in steps of 20 kPa. The driving pressure is measured with a pressure transducer (DRMOD-I2C-R10B, B+B Thermo-Technik GmbH, Germany), and the flow rate of the extruded alginate is measured with a precision scale (DI-100, Denver Instrument, USA).

We then fit the zero-shear viscosity, ${\tilde{\eta }}_{0}$, the corner shear rate, ${\dot{\gamma }}_{c}$, and the power-law shear thinning exponent, *α*, of a 3-parameter Carreau-Yasuda fluid to match the measured flow rate versus pressure relationship. The viscosity-shear rate relationship is given by

$$\begin{array}{c} \tilde{\eta }(\dot{\gamma })={\tilde{\eta }}_{0}[1+(\frac{\dot{\gamma }}{{\dot{\gamma }}_{c}}{)}^{\alpha }{]}^{-1}\;, \end{array}$$ (10)

which is derived from Eq (9) by omitting the infinite-shear viscosity (${\tilde{\eta }}_{\infty }=0$), and introducing the corner-shear rate ${\dot{\gamma }}_{c}=K^{-1}$ as well as the single exponent *α* = *a*_1_ = *a*_2_. Fitting is performed using a Marquard-Levenberg least-squares method implemented in the Python library SciPy, where the squared difference between the measured and the computed flow rate is minimized for each pressure level. The flow rate is computed according to (S-64) with the printing parameters mentioned above and *N* = 150 interpolation intervals between shear rates of 10^−6^ s^−1^ to 10^8^ s^−1^. Since the inner diameter of the printer cartridge is large compared to that of the nozzle, we neglect a possible pressure drop along the cartridge.

### 3.2 Results

**Fig 6:**
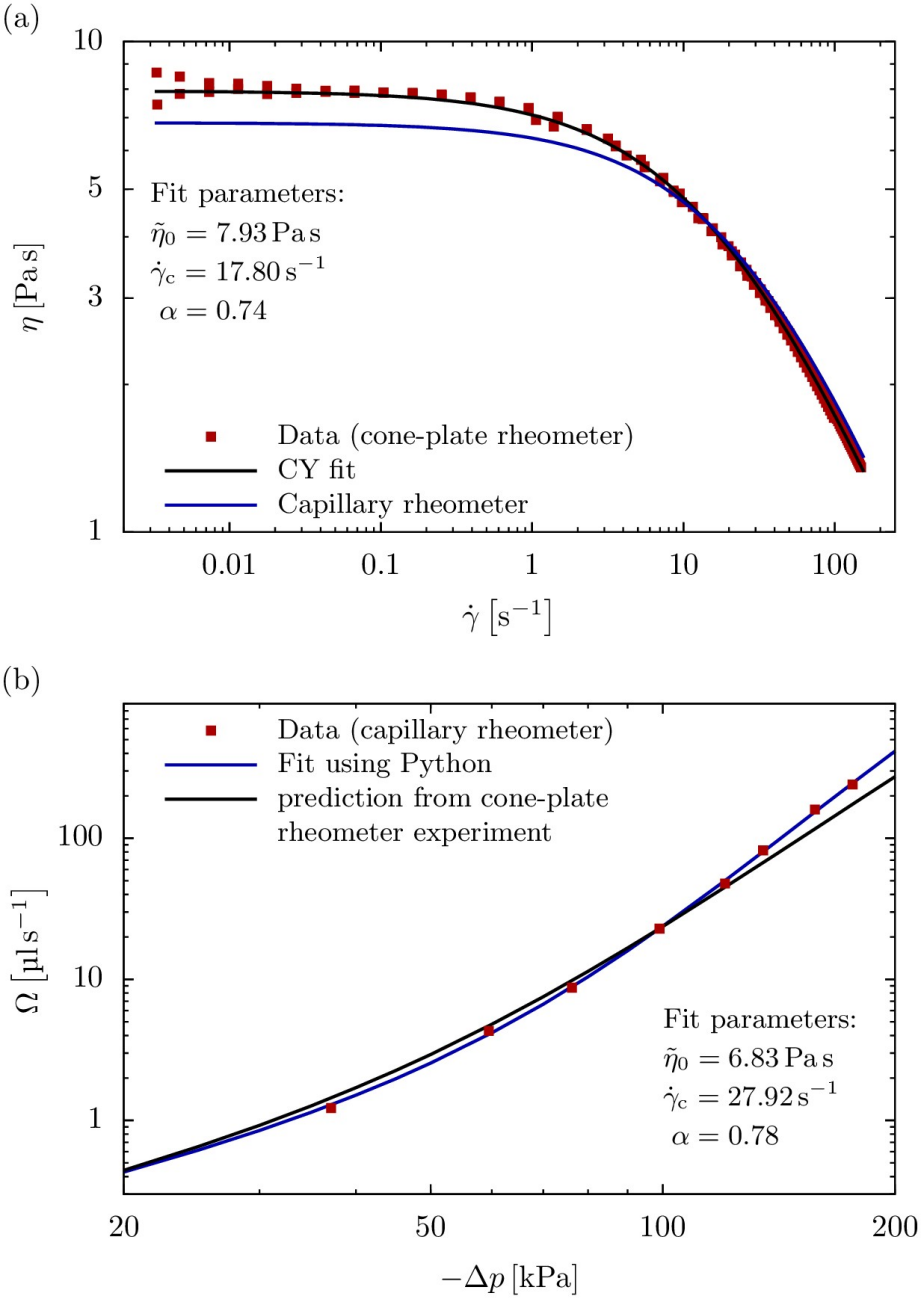
Comparison between capillary rheometer and cone-plate rheometer results. (a) Viscosity versus shear rate for a 2.5% alginate solution as measured with a cone-plate rheometer (data from 4 independent measurements, red squares) shows the pronounced shear thinning of a CY fluid that is well characterized by 3 fit parameters (black line) according to Eq (10). This shear thinning behavior can be predicted (blue line) from an independent capillary rheometry experiment using our Python tool. (b) Flow rate versus pressure relationship of the alginate solution when extruded through a 28 mm long 551 μm diameter capillary (red squares). This relationship follows our numerical solution using 3 fit parameters (blue line). The flow rate versus pressure relationship can similarly be predicted (black line) from the viscosity values obtained from an independent cone-plate rheometer experiment shown in the upper panel, showing significant deviations with increasing pressure.

When measured with a cone-plate rheometer, the viscosity of a 2.5% alginate solution displays a pronounced shear rate dependency (Fig 6a), which is well described by a 3-parameter CY model according to Eq (10). Specifically, at shear rates below the corner shear rate ${\dot{\gamma }}_{c}\approx 17.8\;{\text{s}}^{-1}$, the viscosity is approximately constant, with ${\tilde{\eta }}_{0}\approx 7.9\;\text{Pas}$. At shear rates above ${\dot{\gamma }}_{c}$, the viscosity decreases according to a power-law with exponent *α* ≈ 0.74.

When the same 2.5% alginate solution is extruded through a 28 mm long 551 μm diameter capillary, we find an over-proportional increase in flow rate with increasing pressure (Fig 6b). Specifically, a doubling in pressure causes an approximately 10-fold increase in flow rate. This experimentally measured flow rate versus pressure relationship is exactly predicted by our numerical solution (blue line in Fig 6b), adding further support to the validity of our algorithm.

**Fig 7:**
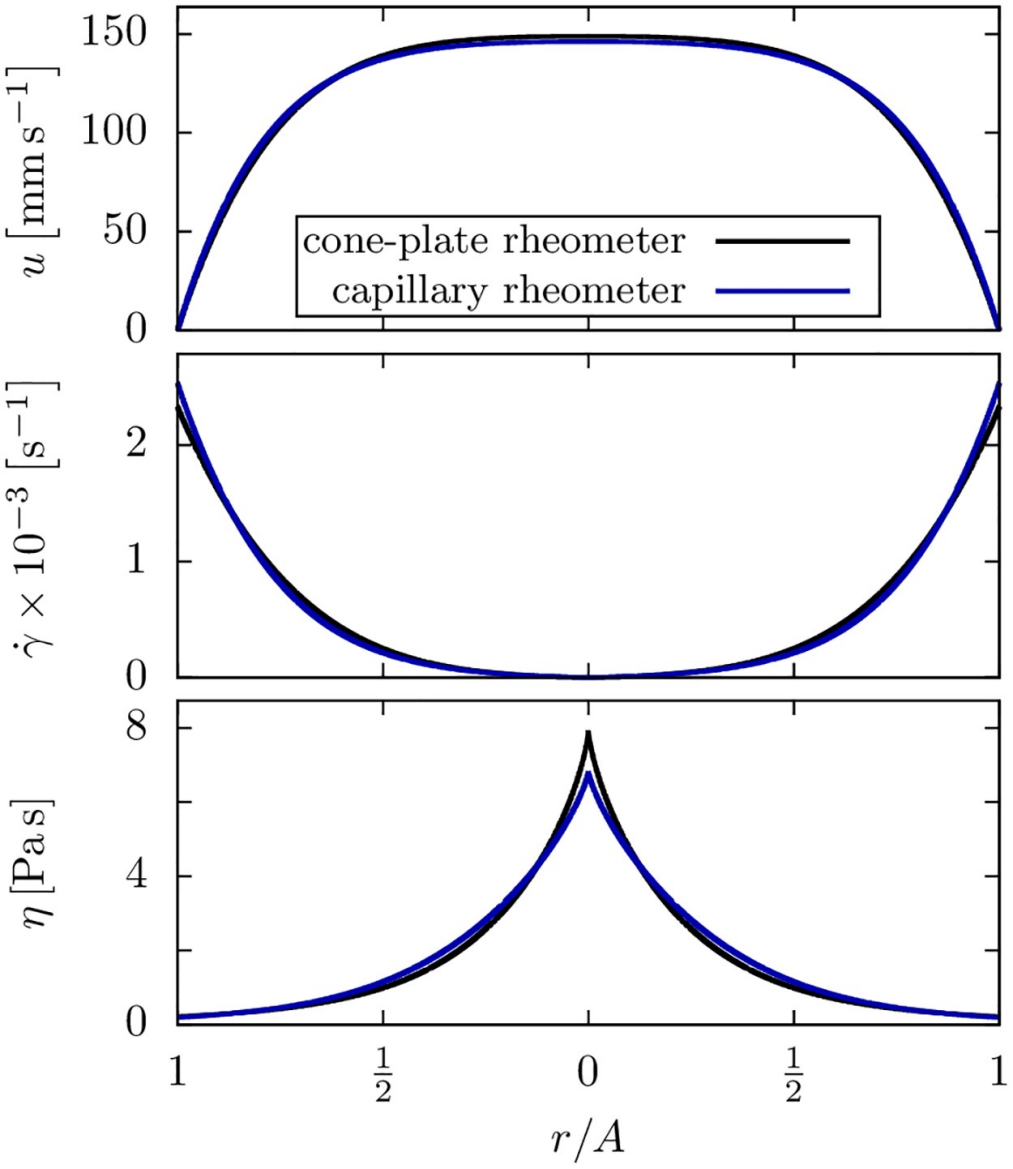
Alginate flow profile from capillary rheometer and cone-plate rheometer data. Flow profiles of 2.5% alginate hydrogel with a pressure difference of Δ*p* = −10^5^ Pa and *N* = 150. There is only a slight difference between the flow profiles calculated from the viscosity parameters obtained with a cone-plate rheometer (black line) and our capillary rheometer (blue line).

If a rheometer is not available, the above procedure can be inverted to obtain the rheological properties of the bioink as follows: starting from a first guess of the CY parameters, the pressure versus flow rate is computed using our Python tool. Subsequently, the viscosity parameters are refined until the prediction matches with the experimental data as shown in Fig 6b. The parameters obtained from the flow-rate versus pressure data (red squares in Fig 6b) are ${\tilde{\eta }}_{0}\approx 6.8\;\text{Pas}$, ${\dot{\gamma }}_{c}\approx 27.9\;{\text{s}}^{-1}$, and *α* ≈ 0.78 and differ only slightly from the parameters extracted from the cone-plate rheometer measurements. Accordingly, also only a slight difference between both parameter sets is seen in the velocity, shear rate and viscosity profiles shown in Fig 7. Also visible in Fig 6b is an increasing deviation of the flow rate versus pressure prediction for the cone-plate rheometer from the measured data with increasing pressure. This is likely due to shear rheometers not being able to achieve the large shear rates that occur under realistic printing conditions, while the capillary rheometer method intrinsically accounts for that.

A specific advantage of this capillary rheometry approach is that the experiment can be performed with the very same bioink that is currently in the printing cartridge prior to the actual printing process. Since most bioprinters are pressure controlled, i.e. the bioink is extruded through a printing needle with a constant pressure, the highly non-linear increase of the flow rate with increasing pressure makes it difficult to find the optimal printing parameters and to predict the material shear stresses during the printing process. Our algorithm solves both problems.

## Conclusion

We presented a simple yet highly accurate algorithm to calculate the velocity and shear rate profiles for generalized Newtonian fluids, such as shear thinning bioinks, in cylindrical nozzles. For this, an arbitrary experimentally known viscosity-shear rate relation is split into a set of continuous intervals described by power-laws. This includes the possibility to predict velocity and shear stress profiles in pure as well as cell-laden bioinks. In each interval, an exact solution for the shear rate and velocity is computed and connected to neighboring intervals to obtain a continuous smooth profile over the entire nozzle diameter. For the shear stress, the linear radial dependency independent of the fluid rheology was confirmed. In addition, the total flow rate as well as the average viscosity, shear rate and shear stress are also found mathematically.

We implemented our method as an easy-to-use Python tool for calculating the velocity and shear rate profiles for a Carreau-Yasuda fluid. To validate this tool, we compared our predictions to a mathematically exact global solution and to Lattice Boltzmann simulations for realistic chitosan hydrogels under typical bioprinting conditions. In both cases, we found excellent agreement. We further measured the velocity profile of an alginate solution in a microfluidic channel and found good agreement with Lattice Boltzmann simulations.

An important experimental application of our theoretical method is capillary rheometry. Here, the flow rate versus pressure relationship for a given hydrogel is obtained using a standard bioprinter. This data can then be fit to our theoretical predictions yielding the corresponding rheological parameters of the bioink. We illustrated this application for alginate and found good agreement with classical rheometer data.

Our method and the accompanying Python implementation provide a fast and simple tool to predict flow rates and shear stresses during bioprinting for a given bioink and thus will help to optimize printing parameters, especially for shear stress-sensitive living cells.

## Supporting information

S1 File Mathematical derivation, further experimental validation, and user’s guide. The supplementary material for the manuscript contains a detailed mathematical derivation of the presented method and a simple model to estimate the force and deformation experienced by a cell in shear thinning capillary flow. We also include further experimental measurements for alginate 2% and 3% and chitosan 3% in square and rectangular microchannels, as well as the corresponding error calculations. A user’s guide for the developed Python tool is provided.
(PDF)

S2 File CYprofiles.py. File containing the implemented classes of our tool.
(PY) Click here for additional data file.

S3 File Tutorial.py. File with a basic usage example for the implemented classes.
(PY) Click here for additional data file.
