# Supplementary material for: Flow and hydrodynamic shear stress inside a printing needle during biofabrication
Source: PLoS One. 2020 Jul 24;15(7):e0236371. doi: 10.1371/journal.pone.0236371 (PMC7380612; doi:10.1371/journal.pone.0236371)
Supplement: S1 File — The supplementary material for the manuscript contains a detailed mathematical derivation of the presented method and a simple model to estimate the force and deformation experienced by a cell in shear thinning capillary flow. We also include further experimental measurements for alginate 2% and 3% and chitosan 3% in square and rectangular microchannels, as well as the corresponding error calculations. A user’s guide for the developed Python tool is provided. (PDF) [file pone.0236371.s001.pdf]

Supplementary material for the manuscript

Flow and hydrodynamic shear stress inside a printing needle during biofabrication

Sebastian J. Müller[1], Elham Mirzahossein[2], Emil Iftekhar[2], Christian Bächer[1], Stefan Schrüfer[3], Dirk W. Schubert[3], Ben Fabry[2] and Stephan Gekle[1]

[1]Biofluid Simulation and Modeling, Universität Bayreuth, Germany
[2]Biophysics Group, Friedrich-Alexander Universität Erlangen-Nürnberg, Germany
[3]Polymer Physics and Processing, Friedrich-Alexander Universität Erlangen-Nürnberg, Germany
Authors to whom correspondence should be addressed:
sjmueller@uni-bayreuth.de, stephan.gekle@uni-bayreuth.de

S-1. Theory

Our algorithm starts from an experimentally known viscosity-shear rate relation $\eta(\dot{\gamma})$ and interpolates it by a series of power-law functions. This interpolation is subsequently used for writing down a similar series of Navier-Stokes equations which are solved first for the shear rate and then for the velocity profile.

S-1.1. Viscosity model

The viscosity-shear rate relationship of the bioink, or any other generalized Newtonian fluid, can be approximated by a continuous, piecewise function

$$\eta\left(\dot{\gamma}\right) = \begin{cases} K_0 \dot{\gamma}^{n_0 - 1} & 0 \leq \dot{\gamma} < \dot{\Gamma}_0 \\ \vdots & \\ K_i \dot{\gamma}^{n_i - 1} & \dot{\Gamma}_{i-1} \leq \dot{\gamma} < \dot{\Gamma}_i \\ \vdots & \\ K_N \dot{\gamma}^{n_N - 1} & \dot{\Gamma}_{N-1} \leq \dot{\gamma} < \infty \end{cases} , \tag{S-1}$$

as depicted in figure 1a of the main text. In every interval the viscosity-shear rate relation is described by a power-law model with a consistency parameter K_i having the physical unit $\mathrm{Pa\,s^{n_i}}$, and a dimensionless exponent n_i, according to the literature [1–7]. We note that the shear rate can also be understood as dimensionless quantity, normalized to a constant shear rate of $1\,\mathrm{s}^{-1}$ without changing its numerical value. Doing so, the consistency parameter can be interpreted as a reference viscosity with the more meaningful physical unit Pa s.
The ith interval is bounded by the shear rates $\dot{\Gamma}_{i-1}$ and $\dot{\Gamma}_i$. The condition

$$K_i \dot{\Gamma}_i^{n_i - 1} = K_{i+1} \dot{\Gamma}_i^{n_{i+1} - 1} \tag{S-2}$$

ensures the continuity of (S-1) across the interval boundary $\dot{\Gamma}_i$ ($i = 0, \ldots, N-1$). Since real fluids usually exhibit Newtonian behavior for zero and infinite shear rates, we take

$$n_0 = n_N = 1 \tag{S-3}$$

for the power-law exponents in the first and last interval.
We note that instead of the power-law interpolation, a linear interpolation would also be possible. However, since most bioinks show power-law shear thinning over a wide range of shear rates, a power-law interpolation is computationally more efficient when logarithmically-spaced shear rate intervals are used, as shown in figure 1a.

S-1.2. Determination of K_i and n_i

Starting from an experimentally known viscosity-shear rate relation $\tilde{\eta}\left(\dot{\gamma}\right)$ which can be given either as raw rheological data or as a viscosity model with known parameters

(e.g. [8, 9]) such as the Carreau-Yasuda model, the consistency indices K_i and exponents n_i in each interpolation interval are determined as follows. The lowest and highest consistency indices are fixed by eq. (S-3) as

$$K_0 = \tilde{\eta}\left(\dot{\Gamma}_0\right) \quad \text{and} \quad K_N = \tilde{\eta}\left(\dot{\Gamma}_{N-1}\right) \quad . \tag{S-4}$$

Since rheological data often spans multiple decades, we choose an equidistant partitioning of the interval $[\dot{\Gamma}_0, \dot{\Gamma}_{N-1}]$ on a logarithmic scale, as shown in figure 1a. Given the bounds of this interval and the number of interpolated points, the intermediate shear rates are given by

$$\dot{\Gamma}_i = \dot{\Gamma}_0 \left(\frac{\dot{\Gamma}_{N-1}}{\dot{\Gamma}_0}\right)^{\frac{i}{N-1}} \quad . \tag{S-5}$$

The parameters of the interpolating power-law functions $\eta_i\left(\dot{\gamma}\right)$ are found by inserting the known viscosity values at the interval bounds. Thus, the following system of equations needs to be solved:

$$K_i \dot{\Gamma}_{i-1}^{n_i-1} = \tilde{\eta}\left(\dot{\Gamma}_{i-1}\right) \tag{S-6}$$

$$K_i \dot{\Gamma}_{i}^{n_i-1} = \tilde{\eta}\left(\dot{\Gamma}_{i}\right) \tag{S-7}$$

By division of the two equations, the power-law exponent is found to be

$$n_i = 1 + \log\left(\frac{\tilde{\eta}\left(\dot{\Gamma}_{i-1}\right)}{\tilde{\eta}\left(\dot{\Gamma}_{i}\right)}\right)\left(\log\left(\frac{\dot{\Gamma}_{i-1}}{\dot{\Gamma}_i}\right)\right)^{-1} \quad . \tag{S-8}$$

Multiplication of (S-6) by (S-7) gives an expression for the consistency index:

$$K_i = \sqrt{\tilde{\eta}\left(\dot{\Gamma}_{i-1}\right)\tilde{\eta}\left(\dot{\Gamma}_{i}\right)}\left(\dot{\Gamma}_{i-1}\dot{\Gamma}_i\right)^{\frac{1-n_i}{2}} \tag{S-9}$$

By inserting a functional form or raw data for $\tilde{\eta}\left(\dot{\gamma}\right)$ into (S-8) and (S-9) the interpolation can be performed in the entire range of shear rates.

S-1.3. Governing equations

The Navier-Stokes equations to determine the flow field $\vec{u}$ read

$$\varrho\left[\frac{\partial \vec{u}}{\partial t} + \left(\vec{u}\cdot\boldsymbol{\nabla}\right)\vec{u}\right] = -\boldsymbol{\nabla} p + \boldsymbol{\nabla}\cdot\left(\underline{\underline{\tau}}\right) + \vec{f} \quad , \tag{S-10}$$

with the fluid mass density ϱ, the pressure gradient $\boldsymbol{\nabla} p$, the viscous stress tensor $\underline{\underline{\tau}}$, and an external force term $\vec{f}$. The viscous stress tensor is related to the viscosity and the strain rate tensor $\underline{\underline{\varepsilon}}$ via

$$\underline{\underline{\tau}} = 2\eta\left(\dot{\gamma}\right)\underline{\underline{\varepsilon}} \quad , \tag{S-11}$$

where the strain rate tensor is defined as

$$\underline{\underline{\varepsilon}} = \tfrac{1}{2}\left[\boldsymbol{\nabla}\vec{u} + (\boldsymbol{\nabla}\vec{u})^{\mathsf{T}}\right] \quad . \tag{S-12}$$

Here, $\boldsymbol{\nabla}\vec{u}$ denotes the dyadic product of the gradient operator and the velocity vector and $(\boldsymbol{\nabla}\vec{u})^{\mathsf{T}}$ its transpose. The shear rate can be obtained as invariant of the strain rate tensor, i. e.

$$\dot{\gamma} = \sqrt{2\sum_{\alpha,\beta}\varepsilon_{\alpha\beta}\varepsilon_{\alpha\beta}} \quad . \tag{S-13}$$

For a purely Newtonian fluid, the viscosity in (S-11) would be a constant.

S-1.3.1. Flow conditions. Analogously to the well-known Poiseuille flow of a Newtonian fluid [10, pp. 180 ff.], we assume a stationary, laminar, and pressure driven flow, with the velocity having only an axial component depending on the radial position. We consider a cylindrical channel and neglect entrance and exit effects. For the following derivation, a cylindrical coordinate system with a radial component r, an azimuthal component ϕ, and an axial component z, is employed. In these coordinates, the flow conditions read:

$$\vec{f} = \vec{0} \tag{S-14}$$

$$\frac{\partial\vec{u}}{\partial t} = \vec{0} \tag{S-15}$$

$$\frac{\partial\vec{u}}{\partial z} = \vec{0} \tag{S-16}$$

$$\frac{\partial\vec{u}}{\partial \phi} = \vec{0} \tag{S-17}$$

$$\vec{u}\,(r,\phi,z) = u\vec{e}_z \tag{S-18}$$

S-1.3.2. Constant pressure gradient. For a purely Newtionan fluid, the flow conditions (S-14)-(S-18) imply a spatially constant pressure gradient throughout the entire channel. In the following, we prove that the same holds for an arbitrary generalized Newtonian fluid. The strain rate tensor in cylindrical coordinates reads:

$$\underline{\underline{\varepsilon}} = \tfrac{1}{2}\begin{pmatrix} \underbrace{2\partial_r u_r}_{\overset{(S-18)}{=}0} & \underbrace{\partial_r u_\phi + \frac{1}{r}\partial_\phi u_r - \frac{1}{r}u_\phi}_{\overset{(S-17),(S-18)}{=}0} & \underbrace{\partial_z u_r}_{\overset{(S-18)}{=}0} + \partial_r u_z \\ - & \underbrace{2\partial_\phi u_\phi + 2\frac{1}{r}u_r}_{\overset{(S-17),(S-18)}{=}0} & \underbrace{\frac{1}{r}\partial_\phi u_z + \partial_z u_\phi}_{\overset{(S-17)}{=}0} \\ - & - & \underbrace{2\partial_z u_z}_{\overset{(S-16)}{=}0} \end{pmatrix} \tag{S-19}$$

where the notation $\partial_x = \frac{\partial}{\partial x}$ denotes a partial spatial derivative with respect to the coordinate x. The "$-$" signs indicate the symmetric components of the tensor. The

underbraced terms vanish due to the flow conditions. Thus, the viscous stress tensor reduces to a single component,

$$\tau_{rz} = \tau_{zr} = \eta(\dot{\gamma}) \frac{\partial u_z}{\partial r} \quad . \tag{S-20}$$

The components of the NSE yield:

$$\frac{\partial p}{\partial r} = 0 \qquad (r-\text{component}) \tag{S-21}$$

$$\frac{\partial p}{\partial \phi} = 0 \qquad (\phi-\text{component}) \tag{S-22}$$

$$\frac{\partial p}{\partial z} = \frac{1}{r}\frac{\partial}{\partial r}(r\tau_{rz}) \qquad (z-\text{component}) \tag{S-23}$$

This shows that the pressure gradient has only a z-component. By applying the derivative ∂_z again on the remaining z-component of the NSE, we obtain:

$$\partial_z^2 p = \partial_z \left[\frac{1}{r}\partial_r (r\eta(\dot{\gamma})\partial_r u)\right] \tag{S-24}$$

$$= \frac{1}{r}\partial_r [r\partial_z (\eta(\dot{\gamma})\partial_r u)] \tag{S-25}$$

$$= \frac{1}{r}\partial_r \left(r\eta(\dot{\gamma})\partial_r \underbrace{\partial_z u}_{\overset{(S-17)}{=}0} + r\partial_r u \underbrace{\partial_z \eta(\dot{\gamma})}_{\overset{(S-17)}{=}0} \right) = 0 \tag{S-26}$$

which shows that the pressure gradient is indeed constant and allows us to define

$$G := \frac{\partial p}{\partial z} = \frac{\Delta p}{L} \quad , \tag{S-27}$$

where $\Delta p = p_L - p_0 < 0$ is the pressure difference along a channel segment of length L. Applying the flow conditions, the Navier-Stokes equations reduce to the ordinary differential equation (1):

$$G = \frac{1}{r}\frac{\partial}{\partial r}\left(r\eta(\dot{\gamma})\frac{\partial u}{\partial r}\right) \tag{S-28}$$

This equation is however still highly non-linear due to the dependency of $\eta(\dot{\gamma})$ on $\partial_r u$ via the shear rate $\dot{\gamma}$ (cf. (S-31), (4)).

S-1.3.3. Ansatz and boundary conditions. Similar to the piecewise viscosity model in (S-1), we decompose the axial velocity $u(r)$ into intervals:

$$u(r) = \begin{cases} u_0(r) & 0 \le r < R_0 \\ \vdots & \\ u_i(r) & R_{i-1} \le r < R_i \\ \vdots & \\ u_N(r) & R_{N-1} \le r < \infty \end{cases} \tag{S-29}$$

as illustrated in figure 1b. The essential difference between (S-1) and (S-29) is that the interval boundaries are determined by shear rates $\dot{\Gamma}_i$ for the former and by radial positions R_i for the latter. While the interval boundaries for the viscosity $\dot{\Gamma}_i$ are an input quantity (see section S-1.2), the radial boundaries are determined *a posteriori* from the shear rate profile by the condition

$$\dot{\gamma}\left(R_i\right) = \dot{\Gamma}_i \tag{S-30}$$

as will be shown in (S-42) below. The shear rate as a function of the radial position $\dot{\gamma}\left(r\right)$ is given by the first derivative of the velocity with respect to the radial position, i. e.

$$\dot{\gamma}\left(r\right) = -\frac{\partial u\left(r\right)}{\partial r} \quad , \tag{S-31}$$

and can also be written in the same piecewise manner:

$$\dot{\gamma}\left(r\right) = \begin{cases} \dot{\gamma}_0\left(r\right) & 0 \leq r < R_0 \\ \vdots & \\ \dot{\gamma}_i\left(r\right) & R_{i-1} \leq r < R_i \\ \vdots & \\ \dot{\gamma}_N\left(r\right) & R_{N-1} \leq r < \infty \end{cases} \tag{S-32}$$

As for classical Poiseuille flow, we assume the common case of the velocity monotonically decreasing with the radial position. According to (S-31), the shear rate is therefore always positive. For $u\left(r\right)$ to be continuously differentiable and finite at the channel center, the piecewise definitions of the velocity and the shear rate must be equal at the intermediate points, R_i, i. e.

$$u_i\left(R_i\right) = u_{i+1}\left(R_i\right) \tag{S-33}$$

and

$$\dot{\gamma}_i\left(R_i\right) = \dot{\gamma}_{i+1}\left(R_i\right) \quad . \tag{S-34}$$

The flow shall further fulfill a no-slip boundary condition at the cylindrical channel wall at $r = A$, i. e.

$$u\left(A\right) = 0 \quad . \tag{S-35}$$

To ensure the continuous differentiability of the axially symmetric flow field, the flow must have a maximum at the channel center, $r = 0$. Therefore, the shear rate has to vanish at this point:

$$\dot{\gamma}\left(0\right) = 0 \tag{S-36}$$

S-1.4. Solution to the shear rate profile

Inserting the ansatz (S-29) and (S-32) into the Navier-Stokes equation (S-28) yields the following system of equations, (3) and (4), where $i \in \{0, \ldots, N\}$ denotes the intervals as above:

$$G = \frac{1}{r} \frac{\partial}{\partial r} \left(-r K_i \dot{\gamma}_i \left(r \right)^{n_i} \right) \tag{S-37}$$

$$\dot{\gamma}_i \left(r \right) = - \frac{\partial u_i \left(r \right)}{\partial r} \quad . \tag{S-38}$$

The first equation (S-37), (3), can be rearranged and integrated once to obtain

$$\dot{\gamma}_i \left(r \right) = \left(- \frac{Gr}{2K_i} - \frac{c_i}{K_i r} \right)^{\frac{1}{n_i}} \quad , \tag{S-39}$$

where the c_i are a set of integration constants that are determined next using the continuity conditions (S-34) and the boundary condition (S-36).

S-1.4.1. Determination of the integration constants of the shear rate profile. The integration constants c_i can be shown to be zero using the complete induction proof described in the following. The base clause, (S-39) for $i = 0$ with the boundary condition (S-36) gives

$$\dot{\gamma}_0 \left(0 \right) = \lim_{r \to 0} \left(- \frac{Gr}{2K_0} - \frac{c_0}{K_0 r} \right)^{\frac{1}{n_0}} \overset{!}{=} 0 \quad , \tag{S-40}$$

which is only fulfilled if the integration constant vanishes, thus, $c_0 = 0$.
Assuming that $c_i = 0$, c_{i+1} can be determined using the continuity condition (S-34),

$$\dot{\gamma}_i \left(R_i \right) = \dot{\gamma}_{i+1} \left(R_i \right) = \dot{\Gamma}_i \quad , \tag{S-41}$$

where the $\dot{\Gamma}_i$ are given. The equality $\dot{\Gamma}_i = \dot{\gamma}_i \left(R_i \right)$ yields an expression for the radial position R_i of the interfacial point between $\dot{\gamma}_i \left(r \right)$ and $\dot{\gamma}_{i+1} \left(r \right)$,

$$R_i = - \frac{2K_i \dot{\Gamma}_i^{n_i}}{G} \quad , \tag{S-42}$$

that can be inserted into the second part of (S-41) using (S-39):

$$\dot{\Gamma}_i = \left(- \frac{GR_i}{2K_{i+1}} - \frac{c_{i+1}}{K_{i+1} R_i} \right)^{\frac{1}{n_{i+1}}} \tag{S-43}$$

$$= \left(\frac{K_i}{K_{i+1}} \dot{\Gamma}_i^{n_i} + \frac{c_{i+1} G}{2K_{i+1} K_i \dot{\Gamma}_i^{n_i}} \right)^{\frac{1}{n_{i+1}}} \tag{S-44}$$

Employing the continuity condition of the viscosity model (S-2) gives an expression for the ratio of the consistency parameters, i. e.

$$K_i \dot{\Gamma}_i^{n_i - 1} = K_{i+1} \dot{\Gamma}_i^{n_{i+1} - 1} \tag{S-45}$$

$$\frac{K_i}{K_{i+1}} = \dot{\Gamma}_i^{n_{i+1} - n_i} \quad . \tag{S-46}$$

Inserting (S-46) into (S-44) finally yields

$$\dot{\Gamma}_i = \left(\dot{\Gamma}_i^{n_{i+1}} + \frac{c_{i+1} G}{2K_{i+1} K_i \dot{\Gamma}_i^{n_i}}\right)^{\frac{1}{n_{i+1}}} \quad , \tag{S-47}$$

which is equal only if $c_{i+1} = 0$ thus completing the proof.
With that, the final form for the shear rate profile in the ith interval is obtained as (cf. (5)):

$$\dot{\gamma}_i\left(r\right) = \left(-\frac{G}{2K_i} r\right)^{\frac{1}{n_i}} \tag{S-48}$$

Note that this solution reduces to the simple power-law model solution if the index i is dropped.

S-1.5. Solution to the velocity profile

The velocity profile is obtained by inserting (S-48), (5), into the second part of the system of differential equations (S-38), (4), and integrating over r:

$$\frac{\partial u_i\left(r\right)}{\partial r} = -\left(-\frac{G}{2K_i} r\right)^{\frac{1}{n_i}} \tag{S-49}$$

$$u_i\left(r\right) = -\left(-\frac{G}{2K_i}\right)^{\frac{1}{n_i}} \frac{n_i}{n_i + 1} r^{1+\frac{1}{n_i}} + \tilde{c}_i \tag{S-50}$$

The integration constants $\tilde{c}_i$ are determined next using the no-slip boundary condition (S-35) and the continuity conditions for the velocity field (S-33).

S-1.5.1. Determination of the integration constants of the velocity profile. Since the number of intervals of the viscosity model N is independent of the choice of the flow parameters, G and A, and this choice uniquely determines the R_i via (S-42), the outer channel boundary R is not necessarily located in the last interval of the velocity ansatz function $u_N\left(r\right)$. Instead, the radius of the channel lies in the kth interval, i. e.

$$R_{k-1} \leq A \leq R_k \quad , \tag{S-51}$$

where $0 < k \leq N$. Intervals with $i > k$ whose boundaries R_i lie beyond the channel radius A have no physical significance and are disregarded in the following. Consequently, the no-slip boundary condition applies to the kth interval:

$$u_k\left(A\right) = -\left(-\frac{G}{2K_k}\right)^{\frac{1}{n_k}} \frac{n_k}{n_k + 1} A^{1+\frac{1}{n_k}} + \tilde{c}_k \stackrel{!}{=} 0 \tag{S-52}$$

The integration constant can therefore easily be found as

$$\tilde{c}_k = \left(-\frac{G}{2K_k}\right)^{\frac{1}{n_k}} \frac{n_k}{n_k + 1} A^{1+\frac{1}{n_k}} \quad . \tag{S-53}$$

For $i < k$, the continuity condition for the velocity field (S-33) can be written as

$$-\left(-\frac{G}{2K_i}\right)^{\frac{1}{n_i}} \frac{n_i}{n_i+1} R_i^{1+\frac{1}{n_i}} + \tilde{c}_i = -\left(-\frac{G}{2K_{i+1}}\right)^{\frac{1}{n_{i+1}}} \frac{n_{i+1}}{n_{i+1}+1} R_i^{1+\frac{1}{n_{i+1}}} + \tilde{c}_{i+1} \quad . \tag{S-54}$$

Assuming that $\tilde{c}_{i+1}$ is known and rearranging this equation for $\tilde{c}_i$ yields

$$\tilde{c}_i = \tilde{c}_{i+1} - R_i \left[\underbrace{\left(-\frac{G}{2K_{i+1}}\right)^{\frac{1}{n_{i+1}}} R_i^{\frac{1}{n_{i+1}}}}_{=\ \dot{\gamma}_{i+1}(R_i)\ =\ \dot{\Gamma}_i} \frac{n_{i+1}}{n_{i+1}+1} - \underbrace{\left(-\frac{G}{2K_i}\right)^{\frac{1}{n_i}} R_i^{\frac{1}{n_i}}}_{=\ \dot{\gamma}_i(R_i)\ =\ \dot{\Gamma}_i} \frac{n_i}{n_i+1} \right] \quad , \tag{S-55}$$

where the underbraced terms can be identified as the shear rates at the interfacial position which are equal by the continuity conditions (S-34). Hence,

$$\tilde{c}_i = \tilde{c}_{i+1} - R_i \dot{\Gamma}_i \left(\frac{n_{i+1}}{n_{i+1}+1} - \frac{n_i}{n_i+1} \right) \quad . \tag{S-56}$$

Finally, inserting the expression for the known outermost integration constant, $\tilde{c}_k$, the interior integration constants can be determined as

$$\tilde{c}_i = \tilde{c}_k - \sum_{j=i}^{k-1} R_j \dot{\Gamma}_j \left(\frac{n_{j+1}}{n_{j+1}+1} - \frac{n_j}{n_j+1} \right) \quad . \tag{S-57}$$

Combining (S-50), (S-53) and (S-57), the velocity profile in the ith interval is given by (6):

$$u_i(r) = -\left(-\frac{G}{2K_i}\right)^{\frac{1}{n_i}} \frac{n_i}{n_i+1} r^{1+\frac{1}{n_i}} + \left(-\frac{G}{2K_k}\right)^{\frac{1}{n_k}} \frac{n_k}{n_k+1} A^{1+\frac{1}{n_k}}$$
$$- \sum_{j=i}^{k-1} R_j \dot{\Gamma}_j \left(\frac{n_{j+1}}{n_{j+1}+1} - \frac{n_j}{n_j+1} \right) \tag{S-58}$$

S-1.6. Calculation of averages

In the following, we derive mathematical expressions for the flow rate as well as the average shear rate, viscosity, and shear stress. The flow rate or, equivalently, the average flow velocity determines the printing speed in 3D bioprinting processes. The average shear rate and shear stress can be used to estimate cell damage during printing [7, 11].

S-1.6.1. Average velocity and flow rate. The cross-sectional average of the velocity field is given by

$$\overline{u} = \frac{1}{\pi A^2} \int_0^{2\pi} \mathrm{d}\phi \int_0^A \mathrm{d}r\, r u(r) \quad . \tag{S-59}$$

The first integral can be evaluated and gives a factor 2π, the second integral is split into the intervals:

$$\overline{u} = \frac{2}{A^2}\left[\int\limits_0^{R_0} \mathrm{d}r\, r u_0(r) + \sum_{i=1}^{k-1} \int\limits_{R_{i-1}}^{R_i} \mathrm{d}r\, r u_i(r) + \int\limits_{R_{k-1}}^{A} \mathrm{d}r\, r u_k(r)\right] \tag{S-60}$$

Abbreviating the prefactors in (S-50), i. e.

$$u_i(r) = a_i r^{1+\frac{1}{n_i}} + \tilde{c}_i \quad , \tag{S-61}$$

with

$$a_i = -\left(-\frac{G}{2K_i}\right)^{\frac{1}{n_i}} \frac{n_i}{n_i+1} \quad , \tag{S-62}$$

the average velocity is found to be:

$$\begin{aligned}\overline{u} = \frac{2}{A^2}\Bigg[&\frac{a_0}{3+\frac{1}{n_0}} R_0^{3+\frac{1}{n_0}} + \frac{\tilde{c}_0}{2} R_0^2 + \sum_{i=1}^{k-1} \frac{a_i}{3+\frac{1}{n_i}}\left(R_i^{3+\frac{1}{n_i}} - R_{i-1}^{3+\frac{1}{n_i}}\right) \\ &+ \sum_{i=1}^{k-1} \frac{\tilde{c}_i}{2}\left(R_i^2 - R_{i-1}^2\right) + \frac{a_k}{3+\frac{1}{n_k}}\left(A^{3+\frac{1}{n_k}} - R_{k-1}^{3+\frac{1}{n_k}}\right) + \frac{\tilde{c}_k}{2}\left(A^2 - R_{k-1}^2\right)\Bigg]\end{aligned} \tag{S-63}$$

The flow rate is given by

$$\Omega = \pi A^2 \overline{u} \quad . \tag{S-64}$$

S-1.6.2. Average shear rate. The same procedure as above can be applied to find the average shear rate. With (S-48) shortened to

$$\dot{\gamma}_i(r) = b_i r^{\frac{1}{n_i}} \quad , \tag{S-65}$$

the average shear rate is given by:

$$\bar{\dot{\gamma}} = \frac{2}{A^2}\left[\frac{b_0}{2+\frac{1}{n_0}} R_0^{2+\frac{1}{n_0}} + \sum_{i=1}^{k-1} \frac{b_i}{2+\frac{1}{n_i}}\left(R_i^{2+\frac{1}{n_i}} - R_{i-1}^{2+\frac{1}{n_i}}\right) + \frac{b_k}{2+\frac{1}{n_k}}\left(A^{2+\frac{1}{n_k}} - R_{k-1}^{2+\frac{1}{n_k}}\right)\right] \tag{S-66}$$

S-1.6.3. Average viscosity. The viscosity field, $\eta(r)$, is calculated by inserting the shear rate field from (S-48) into the power-law definitions of the respective interpolation interval in (S-1). Thus,

$$\eta_i(r) = K_i\left(\dot{\gamma}_i(r)\right)^{n_i-1} = K_i\left(-\frac{G}{2K_i} r\right)^{1-\frac{1}{n_i}} \quad , \tag{S-67}$$

which can be shortened to

$$\eta_i(r) = d_i r^{1-\frac{1}{n_i}} \quad . \tag{S-68}$$

Using the same procedure as above, the integral in the ith interval yields

$$\int_{R_{i-1}}^{R_i} \mathrm{d}r\, r\eta_i(r) = \frac{d_i}{3-\frac{1}{n_i}}\left(R_i^{3-\frac{1}{n_i}} - R_{i-1}^{3-\frac{1}{n_i}}\right) \tag{S-69}$$

if $n_i \neq \frac{1}{3}$ and

$$\int_{R_{i-1}}^{R_i} \mathrm{d}r\, r\eta_i(r) = d_i \int_{R_{i-1}}^{R_i} \mathrm{d}r\, r^{-1} = d_i \ln\left(\frac{R_i}{R_{i-1}}\right) \tag{S-70}$$

if $n_i = \frac{1}{3}$. The average viscosity for our model is therefore given by

$$\overline{\eta} = \frac{2}{A^2}\left[\int_0^{R_0} \mathrm{d}r\, r\eta_0(r) + \sum_{i=1}^{k-1}\int_{R_{i-1}}^{R_i} \mathrm{d}r\, r\eta_i(r) + \int_{R_{k-1}}^{A} \mathrm{d}r\, r\eta_k(r)\right] \quad , \tag{S-71}$$

where the integrals are chosen as (S-69) or (S-70) according to n_i. ‡

S-1.6.4. Average shear stress. The radial profile of the shear stress is given as the product of the shear rate field (S-48) and the viscosity field. The latter is calculated by inserting the shear rate field from (S-48) into the power-law definitions of the respective interpolation interval in (S-1):

$$\eta_i(r) = K_i(\dot{\gamma}_i(r))^{n_i - 1} \tag{S-72}$$

The shear stress profile is therefore obtained as

$$\begin{aligned}\sigma_i(r) &= \dot{\gamma}_i(r)\,\eta_i(r) = K_i(\dot{\gamma}_i(r))^{n_i} \\ &= -\tfrac{1}{2}Gr = \sigma(r) \quad ,\end{aligned} \tag{S-73}$$

where the index i can be dropped since it is independent of the viscosity interpolation. This linear relationship of shear stress and radial position is well-known for power-law fluids [11]. Its average is found by simply solving one integral that yields:

$$\overline{\sigma} = \frac{2}{A^2}\int_0^A \mathrm{d}r\, r\sigma(r) = -\frac{GA}{3} \tag{S-74}$$

‡ Note that in the inner-most interval with R_0 as its right boundary the shear rate is always close to zero and thus the fluid is Newtonian with $n_0 = 1$ (see (S-3)) such that the mathematically undefined situation of (S-70) with $R_{i-1} = 0$ is excluded.

S-1.7. Global analytical solution for a simplified CY model

In order to validate the algorithm presented above according to the procedure described in section 2 of the manuscript, we calculate a global mathematical solution to the Navier-Stokes equation (S-28), (1), for a simplified Carreau-Yasuda model. In the following, we derive an analytical solution for the flow profiles of a CY model (cf. (9)) with the following simplification:

$$\begin{aligned} \eta_\infty &= 0 \\ a_1 = a_2 &= 1 \end{aligned} \tag{S-75}$$

The viscosity as a function of the shear rate is therefore given as (cf. (8))

$$\tilde{\eta}\left(\dot{\gamma}\right) = \frac{\eta_0}{1 + K\dot{\gamma}} \quad . \tag{S-76}$$

Using the same assumptions as in section S-1.3.1, the NSE yields:

$$G = \frac{1}{r}\frac{\partial}{\partial r}\left(-r\frac{\eta_0\dot{\gamma}}{1 + K\dot{\gamma}}\right) \tag{S-77}$$

After a first integration and rearrangement of the equations one obtains

$$\dot{\gamma}\left(r\right) = \frac{-\frac{Gr}{2} - \frac{c_1}{r}}{\eta_0 + \frac{KGr}{2} + \frac{Kc_1}{r}} \tag{S-78}$$

and application of boundary condition (S-36) determines the integration constant as $c_1 = 0$. Therefore, the shear rate profile is given as:

$$\dot{\gamma}\left(r\right) = \frac{-\frac{Gr}{2}}{\eta_0 + \frac{KGr}{2}} = -\frac{1}{\frac{2\eta_0}{Gr} + K} \tag{S-79}$$

Inserting this result into (S-38) and integrating the resulting equation gives the velocity profile:

$$\begin{aligned} u\left(r\right) &= -\int \mathrm{d}r\,\dot{\gamma}\left(r\right) \\ &= \frac{r}{K} - \frac{2\eta_0}{GK^2}\ln\left(\frac{2\eta_0}{G} + Kr\right) + c_2 \end{aligned} \tag{S-80}$$

The second integration is easily found by applying the no-slip boundary condition (S-35). Thus, the velocity profile is given by:

$$u\left(r\right) = \frac{r - A}{K} + \frac{2\eta_0}{GK^2}\ln\left(\frac{\frac{2\eta_0}{G} + KA}{\frac{2\eta_0}{G} + Kr}\right) \tag{S-81}$$

S-1.8. Estimate of force and deformation experienced by flowing cells

In this section, we provide a simple approach to estimate the force sensed by a cell and its resulting deformation during printing.
Both quantities depend on the radial position r at which the cell transitions through the channel. Considering, in a first approximation, the cell as a sphere with radius R_c, the shear force acting on it is given by the surface integral of the shear stress over the sphere. Due to the linearity of the shear stress with the radial position, and as long the cell is small compared to the channel, the force is obtained as the product of the surface area and the shear stress at the radial position of the sphere center, as detailed next.
With the shear stress given in (S-73), the shear force acting on the cell is obtained as:

$$
\begin{aligned}
F_z &= \int_0^{2\pi}\int_0^{\pi} \sigma\left(R_{\text{surface}}\right) R_c^2 \sin\theta \,\mathrm{d}\theta \;\mathrm{d}\phi \; = 2\pi R_c^2 \int_0^{\pi} \sigma\left(r + R_c\cos\theta\right)\sin\theta\,\mathrm{d}\theta \\
&= 2\pi R_c^2\left(-\frac{G}{2}\right)\left[\underbrace{\int_0^{\pi} r\sin\theta\,\mathrm{d}\theta}_{=2r} + \underbrace{\int_0^{\pi} R_c\cos\theta\sin\theta\,\mathrm{d}\theta}_{=0}\right] = 4\pi R_c^2\left(-\frac{G}{2}r\right) \\
&= A_{\text{cell}}\cdot\sigma\left(r\right)
\end{aligned}
\tag{S-82}
$$

The result is depicted in figure S-1a.
Another critical quantity is the cell deformation, which can be approximated using the shear stress and the mechanical properties of the cell. As a rough estimate, we assume the cell to behave linearly elastic with the stress–strain relationship given as

$$
\varepsilon\left(r\right) = \frac{\sigma\left(r\right)}{E} \quad , \tag{S-83}
$$

where the strain ε quantifies the relative stretching of the cell. The Young's modulus E is chosen as 1 kPa to 10 kPa to cover the typical range of stiffness for cells [13]. This leads to significant deformations as shown in figure S-1b which reiterates the importance of hydrodynamic shear forces in bioprinting.

Figure S-1. (a) The force F acting on cells as radial function according to (S-82). (b) The strain according to (S-83) as a deformation measure for a linear elastic cell. The corresponding flow profile is shown in fig. 3. We note that the assumption of linear elasticity predicts relatively large deformations, which would not be the case for a more realistic, strain-hardening behavior.

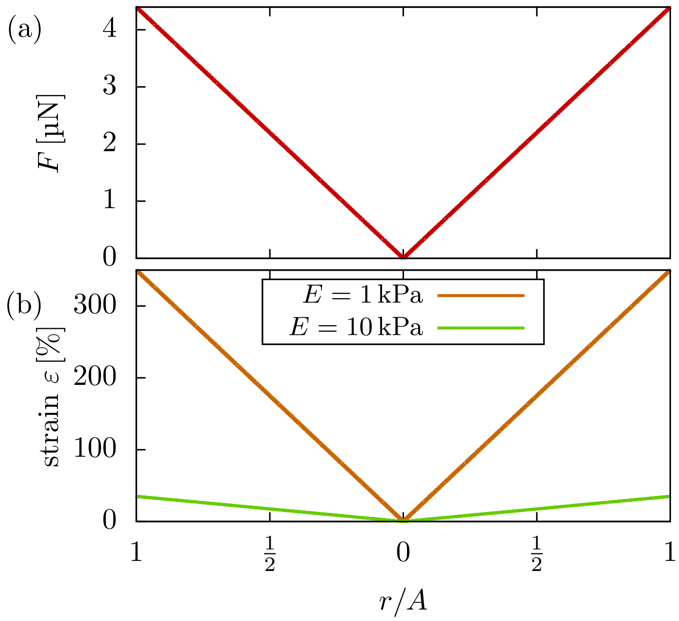

S-1.9. Inclusion of wall-slip effects

Wall-slip effects are sometimes reported, especially for fluids exhibiting non-Newtonian behavior [12] or highly hydrophobic channel coatings. The general approach to include a velocity slip at a wall is to allow for a finite tangential velocity at this point or, equivalently, to shift (in the calculations) the channel wall further outwards by a distance known as the slip length. It is thus straightforward to incorporate slip effects into our calculations if the slip length is known.
Alternatively, if the slip velocity next to the wall is known instead of the slip length, simply shifting upwards the computed no-slip velocity profile by a constant value represents a very good approximation. The shear rate would stay unchanged, and likewise the viscosity and the shear stress. Therefore, the inclusion of slip effects in our algorithm would be unproblematic.

S-2. Additional experimental validation of the algorithm

S-2.1. Additional experiments

In this section, we provide more validation to our algorithm with experimental measurements. Using the same approach as detailed in 2.3 of the manuscript, we performed velocity profile measurements of 2 % alginate at 100, 200, and 300 kPa, of 3 % alginate at 300, 400, and 500 kPa, and 3 % chitosan at 300, and 400 kPa. These measurements as well as the velocity profiles calculated using our Lattice Boltzmann method are depicted in figure S-2, showing good agreement. Since the pressure drop in the connectors and tubings is unknown but depends on the rheology of the hydrogel, we assume a constant pressure drop before the microchannel of 21 % for 2 % alginate, 10 % for 3 % alginate, and 23 % for 3 % chitosan, respectively. Additionally, we measured the flow profile of 3 % alginate in a rectangular microchannel with 1000 µm × 200 µm cross section. Due to limitations of the field of view of the microscope, less than one half of the channel could be focused during the measurements. In figure S-3, the mirrored experimentally measured velocity profile is shown in comparison to our Lattice Boltzmann calculations. Due to connectors and tubing with a diameter of approximately the size of the microchannel, the pressure drop of 48 % is reasonable.

S-2.2. Error quantification

In the following, we present an error calculation for the different flow experiments. First, we calculate an averaged velocity profile for the measurements as well as our calculations by averaging the data in a given d-interval. For the square channel, we choose a bin width of $d_{\mathrm{bin}} = 7.31\,\mu\mathrm{m}$ (corresponding to $N_{\mathrm{bins}} = 26$ bins in the range of $d = -95\,\mu\mathrm{m}$ to $95\,\mu\mathrm{m}$), for the rectangular channel $d_{\mathrm{bin}} = 16.3\,\mu\mathrm{m}$ (corresponding to $N_{\mathrm{bins}} = 26$ bins

Figure S-2. Experimentally measured velocity profiles in a 190 µm × 190 µm microchannel in comparison to numerical results using the Lattice Boltzmann method. (a, c, e) 2 % alginate at 100, 200, and 300 kPa. (b, d, f) 3 % alginate at 300, 400, and 500 kPa. (g, h) 3 % chitosan at 300 and 400 kPa.

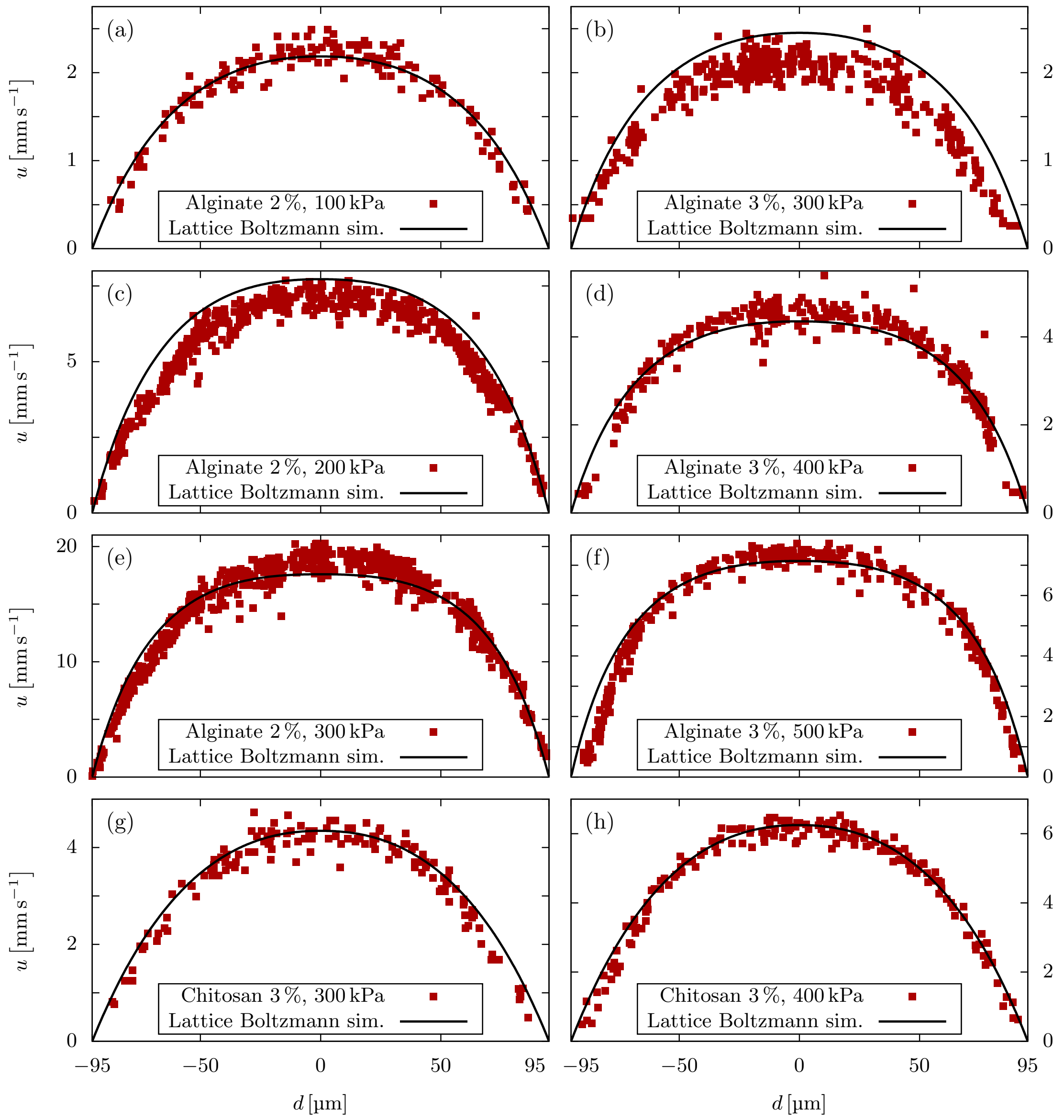

in the range of $d = 75\,\mu\text{m}$ to $500\,\mu\text{m}$). The average velocity in each bin is computed by

$$\overline{u}_i = \frac{1}{N_i} \sum_{k=1}^{N_i} u_k \quad , \tag{S-84}$$

Figure S-3. Experimentally measured velocity profiles of 3 % alginate at 400 and 500 kPa in a 1 mm × 200 µm microchannel in comparison to numerical results using the Lattice Boltzmann method. The experimental data is mirrored with respect to the channel center.

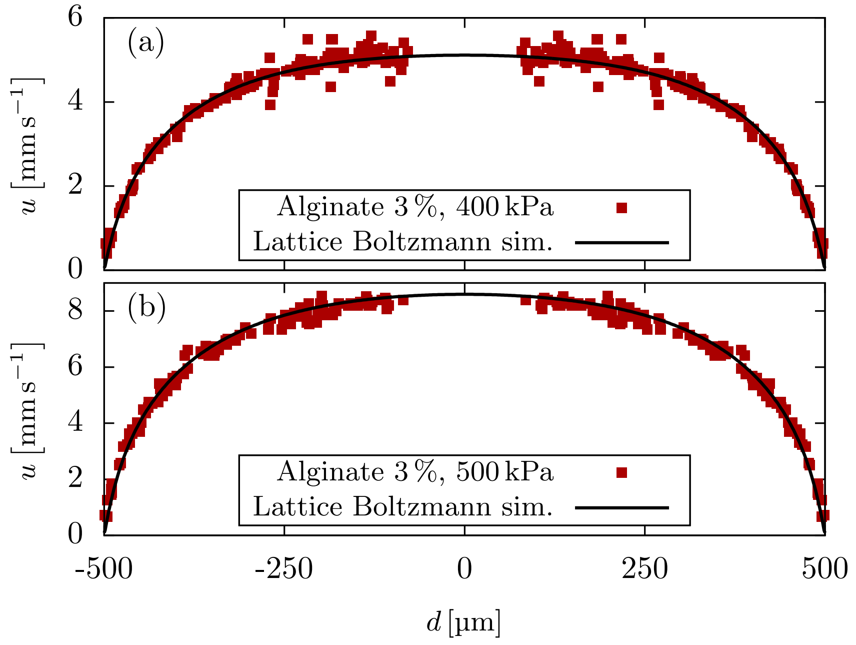

where N_i is the number of data points in the i-th bin. Using this approach, we additionally calculate the standard deviation of the data from the average value as

$$\sigma_{u,i} = \left(\frac{1}{N_i - 1} \sum_{k=1}^{N_i} (u_k - \overline{u}_i)^2 \right)^{\frac{1}{2}} \quad . \tag{S-85}$$

Using the averaged profiles, we calculate the relative error between measurement and Lattice Boltzmann computation as:

$$\epsilon = \left(\frac{1}{N_{\text{bins}}} \sum_{k=1}^{N_{\text{bins}}} \frac{\left(\overline{u}_k^{\text{Exp}} - \overline{u}_k^{\text{LB}} \right)^2}{\left(\overline{u}_{\text{max}}^{\text{LB}} \right)^2} \right)^{\frac{1}{2}} \tag{S-86}$$

The averaged profiles with a range of $\pm\sigma_{u,i}$ are shown in figure S-4 for the square microchannel and in figure S-5 for the rectangular microchannel, where we find relative errors in the range of $\epsilon = 3.3\,\%$ to $13.5\,\%$, and $\epsilon = 2\,\%$, respectively.

Figure S-4. Averaged profiles from figure S-2. The gray area indicates the range of one standard deviation from the mean curve. ϵ gives the relative error calculated according to (S-86).

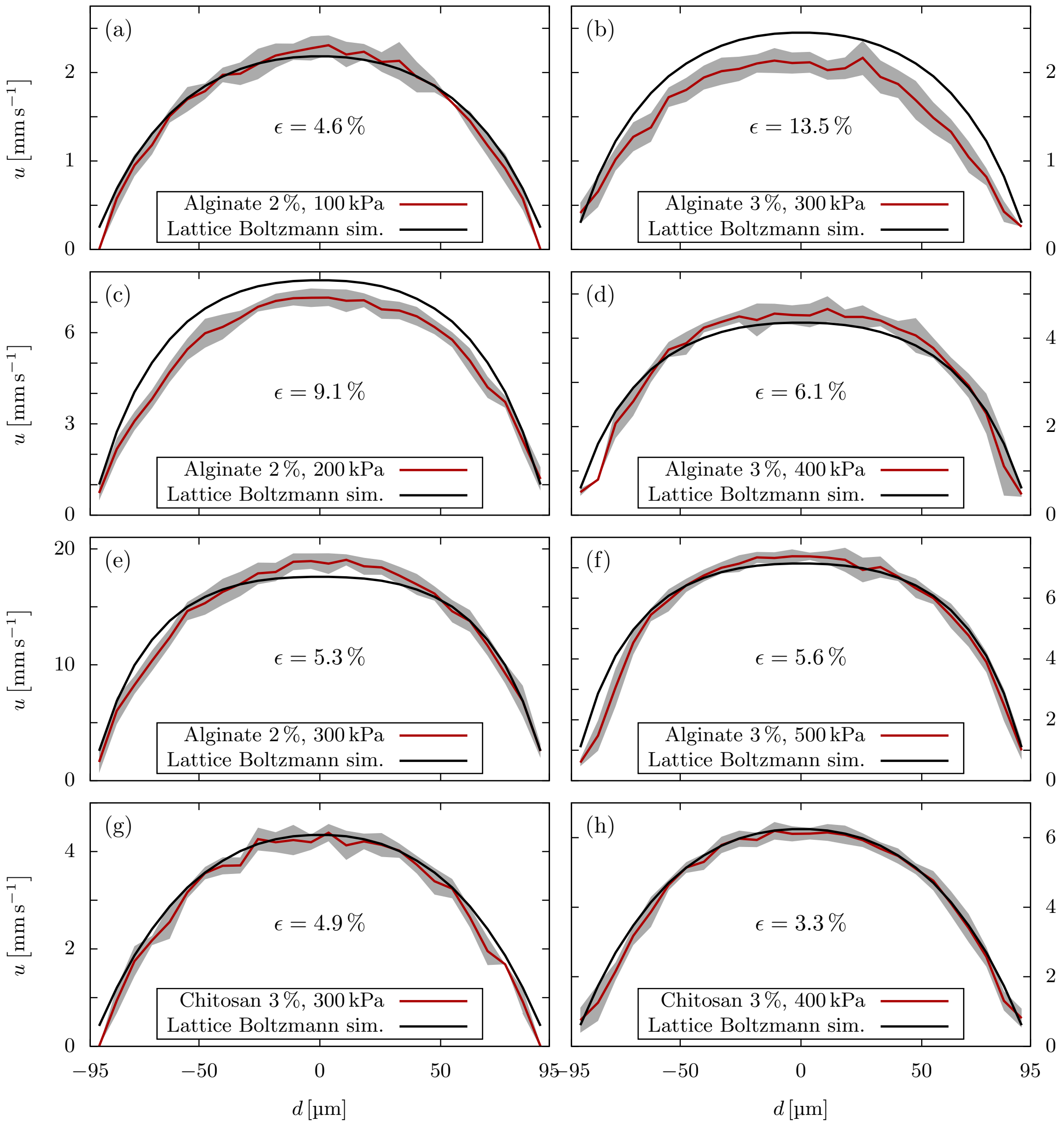

Figure S-5. Averaged profiles from figure S-3. The gray area indicates the range of one standard deviation from the mean curve. ϵ gives the relative error calculated according to (S-86).

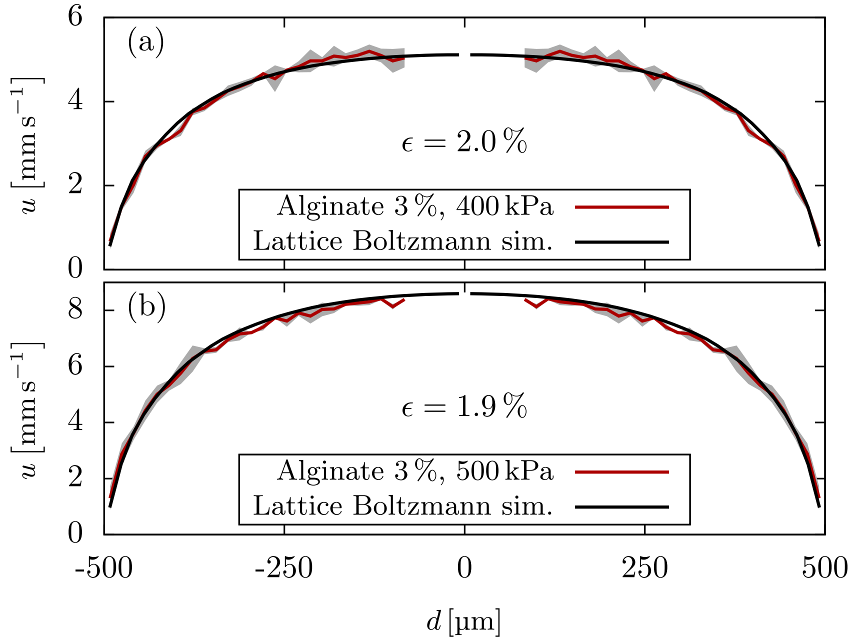

S-3. Computational procedure and user guide

This section describes the structure and usage of the Python classes, found in the Supplementary Material, that implement the presented algorithm for the Carreau-Yasuda model. The first part gives an overview of the four implemented Python classes. The second part provides a short user guide explaining how to use the classes for flow profile calculations. Note that their usage requires a Python (version 2 or 3) installation [14, 15]. The use of a Python IDE, e. g. Spyder [16], Thonny [17] or PyCharm [18], is optional but can be advantageous. The coloring in the code examples is the following: `blue` denotes classes, `green` denotes variables, and `gray` means a comment.
For the Carreau-Yasuda model [19] the viscosity is given by

$$\tilde{\eta}\left(\dot{\gamma}\right) = \tilde{\eta}_\infty + \frac{\tilde{\eta}_0 - \tilde{\eta}_\infty}{\left[1 + (K\dot{\gamma})^{a_1}\right]^{\frac{a_2}{a_1}}} \quad , \tag{S-87}$$

where $\tilde{\eta}_0$ and $\tilde{\eta}_\infty$ are the viscosities in the limit of zero and infinite shear rates and K is a time constant with the unit s. Its inverse, $K^{-1} = \dot{\gamma}_c$, is sometimes referred to as *corner shear rate* and determines the transition to the zero-shear Newtonian plateau. The exponents a_1 and a_2 determine the shape of the transition between the zero-shear Newtonian plateau and the power-law region as well as the power-law behavior.

S-3.1. Overview of Python classes

The tool uses four classes that hold the input parameters, perform the calculations and save or plot output data. They can be found in the file `CYprofiles.py`. The four classes are:

(i) `Analytical_Viscosity()`: instances of this class hold the parameters of the Carreau-Yasuda model and can calculate the viscosity for a given shear rate according to (S-87).

(ii) `Interpolation()`: instances of this class perform the interpolation of a given `Analytical_Viscosity()` in a provided range of shear rates using the partitioning described in section S-1.2.

(iii) `Printing_Parameters()`: instances of this class hold the printing parameters, i.e. the nozzle radius and the pressure gradient or the flow rate.

(iv) `Profiles()`: instances of this class perform the calculation of the velocity, shear rate, and viscosity profile for given `Interpolation()` and `Printing_Parameters()` according to the presented algorithm. If a flow rate is provided, the corresponding pressure gradient is calculated iteratively to match the given flow rate.

S-3.2. User guide

This section is meant to serve as an explanatory tutorial for our Python tool. It will cover the two main steps necessary for calculating a flow profile: the viscosity

interpolation according to section S-1.2 and the profile calculation according to the presented algorithm. The following code examples can be found in the file `tutorial.py`.

Step 1 - Performing the interpolation Starting point for the viscosity interpolation is the Carreau-Yasuda model. After fitting rheological data, the values of its parameters in (S-87) are known. The `Analytical_Viscosity()` is then initialized by:

```
# initialize variables
eta0     =  1.0e2   ≙  η̃0     [Pa s]
etainf   =  1.0e-3  ≙  η̃∞     [Pa s]
K        =  1.0e-3  ≙  K      [s]
a1       =  0.3     ≙  a1
a2       =  0.9     ≙  a2

# initialize Analytical_Viscosity instance
analytical = Analytical_Viscosity( eta0=eta0, etainf=etainf,K=K,a1=a1,a2=a2 )
```

To perform the interpolation, the range of shear rates to interpolate and the number of (power-law) intervals is required:

```
# initialize variables
gamma0      =  1.0e-6  ≙  Γ̇0       [s^-1]
gammaN      =  1.0e6   ≙  Γ̇N-1     [s^-1]
Ninterpol   =  100     ≙  N - 1

# initialize Interpolation instance
interpol= Interpolation(gamma0=gamma0, gammaN=gammaN,
  Ninterpol=Ninterpol, analytical=analytical)
```

The calculation is then simply performed by executing

```
interpol.calculate_interpolation()
```

and the interpolation can be checked by plotting the calculated data via

```
interpol.plot_interpolation()
```
.

To save the viscosity interpolation, one can use

```
interpol.save_interpolation(file)
```

to save the data in viscosity-shear rate format and

```
interpol.save_interpolation_parameters(file)
```

to save the power-law parameters for all intervals in a file.

Step 2 - Calculating the flow profile Once the interpolation is completed, the next step is the definition of the printing parameters, i. e. the channel radius and the pressure gradient. This is done by:

```
# initialize variables
rchannel  =  1.0e-4  ≙  A  [m]
pgrad     =  -1.0e7  ≙  G  [Pa m^-1]

# initialize Printing_Parameters instance
printparams = Printing_Parameters(
  pressureGradient=pgrad, channelRadius=rchannel)
```

To calculate the radial profiles for the velocity (in $\mathrm{m\,s^{-1}}$), the shear rate (in $\mathrm{s^{-1}}$), and the viscosity (in Pa s), the `Profiles()` class is initialized with printing parameters and an interpolation by

```
# initialize Profiles instance
fluidprofiles = Profiles(
  interpolation=interpol, printingParameters=printparams)
```

Finally, the calculation is performed by executing

```
fluidprofiles.calculate_profiles()
```

and the data can be plotted using the following methods:

```
fluidprofiles.plot_velocity()
fluidprofiles.plot_shearrate()
fluidprofiles.plot_shearstress()
fluidprofiles.plot_viscosity()
```

The data for all calculated fields is saved to a file with

```
fluidprofiles.save_profiles(file)
```

and

```
fluidprofiles.save_averages(file)
```

for the averaged quantities, respectively.
In the case of an imposed flow rate, i. e. if the pressure gradient is unknown, our tool automatically computes the corresponding pressure gradient necessary for the profile calculation. To do so, solely the initialization of the printing parameters changes as follows:

```
# initialize variables
rchannel  =  1.0e-4  ≙  A  [m]
flowrate  =  1.0e-9  ≙  Ω  [m^3 s^-1]
```

```
# initialize Printing_Parameters instance
printparams = Printing_Parameters(
  flowrate=flowrate, channelRadius=rchannel)
```

S-4. Lattice Boltzmann algorithm for generalized Newtonian fluids

This section briefly summarizes the Lattice Boltzmann method and the extension we added to the open-source package ESPResSo [20]. For an introduction into the Lattice Boltzmann method we refer the interested reader to the book by Krüger et al.[21]. The Lattice Boltzmann equation for the multiple relaxation time scheme used in ESPResSo reads:

$$f_i\left(\vec{x}+\vec{c}_i\Delta t, t+\Delta t\right) - f_i\left(\vec{x},t\right) = \sum_{j=0}^{18}\left(\underline{\underline{M}}^{-1}\underline{\underline{\omega}}\,\underline{\underline{M}}\right)_{ij}\left(f_j\left(\vec{x},t\right) - f_i^{\mathrm{eq}}\left(\vec{x},t\right)\right) \tag{S-88}$$

It describes the collision and streaming of the population distribution f_i $(i = 0, \ldots, 18)$ during one time step Δt. Here, $\vec{c}_i$ are the discretized lattice velocities, $\underline{\underline{M}}$ denotes transformation matrix that maps the populations onto moment space, $\underline{\underline{\omega}}$ is the diagonal relaxation frequency matrix, and f_i^{eq} denote the equilibrium population distributions. The relaxation frequency for the shear moments ω_{S} is related to the dynamic viscosity of the fluid via [22]

$$\eta = \varrho c_s^2\left(\frac{1}{\omega_{\mathrm{S}}} - \tfrac{1}{2}\right)\Delta t \quad , \tag{S-89}$$

with the fluid mass density ϱ and the lattice speed of sound c_s. The calculation of the viscosity according to the rheological model requires the local shear rate at each lattice node. Chai *et al* [22] showed that the local strain rate tensor can be obtained from the populations by

$$\varepsilon_{\alpha\beta} = -\frac{1}{2\varrho c_s^2\Delta t}\sum_{i,j=0}^{18}\left[\left(\vec{c}_i\right)_\alpha\left(\vec{c}_i\right)_\beta\left(\underline{\underline{M}}^{-1}\underline{\underline{\omega M}}\right)_{ij}\left(f_j\left(\vec{x},t\right) - f_i^{\mathrm{eq}}\left(\vec{x},t\right)\right)\right] \quad . \tag{S-90}$$

The shear rate is then obtained as invariant of the strain rate tensor according to (S-13). From the local shear rate, the viscosity according to the rheological model and the local relaxation time according to (S-89) are computed at each lattice node and updated in every time step.

In order to ensure simulation stability, we choose the time step globally according to Krüger *et al* [21, p. 273] as

$$\Delta t = c_s^2\left(\tau - \tfrac{1}{2}\right)\frac{\Delta x^2}{\nu^\star} = \frac{\Delta x^2}{6\nu^\star} \quad , \tag{S-91}$$

with $c_s^2 = \frac{1}{3}$, a global relaxation parameter $\tau = 1$, and a reference kinematic viscosity $\nu^\star$. The latter is provided, for instance, by the upper Newtonian viscosity plateau of

the corresponding CY model.
At the boundary of the cylindrical channel a bounce-back algorithm is applied to realize a no-slip boundary condition. The flow is driven by a pressure gradient along the z-direction, which is realized as external force density in the algorithm.

References

[1] Blaeser A, Duarte Campos D F, Puster U, Richtering W, Stevens M M and Fischer H 2015 *Advanced Healthcare Materials* **5** 326–333 ISSN 2192-2640

[2] Fu T, Carrier O, Funfschilling D, Ma Y and Li H Z 2016 *Chemical Engineering & Technology* **39** 987–992 ISSN 09307516 URL http://doi.wiley.com/10.1002/ceat.201500620

[3] Fyrippi I, Owen I and Escudier M 2004 *Flow Measurement and Instrumentation* **15** 131–138 ISSN 0955-5986

[4] Ning L, Betancourt N, Schreyer D J and Chen X 2018 *ACS Biomaterials Science & Engineering* **4** 3906–3918 ISSN 2373-9878, 2373-9878 URL http://pubs.acs.org/doi/10.1021/acsbiomaterials.8b00714

[5] Ruschak K J and Weinstein S J 2014 *Polymer Engineering & Science* **54** 2301–2309 ISSN 00323888 URL http://doi.wiley.com/10.1002/pen.23782

[6] Tian X Y, Li M G, Cao N, Li J W and Chen X B 2009 *Biofabrication* **1** 045005 ISSN 1758-5082

[7] Snyder J, Rin Son A, Hamid Q, Wang C, Lui Y and Sun W 2015 *Biofabrication* **7** 044106–17

[8] Escudier M, Gouldson I, Pereira A, Pinho F and Poole R 2001 *Journal of Non-Newtonian Fluid Mechanics* **97** 99–124 ISSN 03770257 URL http://linkinghub.elsevier.com/retrieve/pii/S0377025700001786

[9] Wu Q, Therriault D and Heuzey M C 2018 *ACS Biomaterials Science & Engineering* **4** 2643–2652 ISSN 2373-9878, 2373-9878 URL http://pubs.acs.org/doi/10.1021/acsbiomaterials.8b00415

[10] Batchelor G K 2000 *An Introduction to Fluid Dynamics* (Cambridge: Cambridge University Press) ISBN 978-0-511-80095-5 URL http://ebooks.cambridge.org/ref/id/CBO9780511800955

[11] Paxton N, Smolan W, Böck T, Melchels F, Groll J and Jüngst T 2017 *Biofabrication* **9** 044107 ISSN 1758-5090

[12] Sarker M and Chen X B 2017 *Journal of Manufacturing Science and Engineering* **139** 081002 ISSN 1087-1357, 1528-8935

[13] Yokokura T, Nakashima Y, Yonemoto Y, Hikichi Y and Nakanishi Y 2017 *International Journal of Engineering Science* **114** 41–48 ISSN 00207225

[14] Oliphant T E 2007 *Computing in Science & Engineering* **9** 10–20 ISSN 1521-9615

[15] 2019 Welcome to Python.org URL https://www.python.org/

[16] 2019 Spyder Website URL https://www.spyder-ide.org/

[17] 2019 Thonny, Python IDE for beginners URL https://thonny.org/

[18] 2019 PyCharm: the Python IDE for Professional Developers by JetBrains URL https://www.jetbrains.com/pycharm/

[19] Carreau P J 1972 *Transactions of the Society of Rheology* **16** 99–127 ISSN 0038-0032 URL http://sor.scitation.org/doi/10.1122/1.549276

[20] Limbach H, Arnold A, Mann B and Holm C 2006 *Computer Physics Communications* **174** 704–727 ISSN 00104655 URL https://linkinghub.elsevier.com/retrieve/pii/S001046550500576X

[21] Krüger T, Kusumaatmaja H, Kuzmin A, Shardt O, Silva G and Viggen E M 2017 *The Lattice Boltzmann Method* Graduate Texts in Physics (Cham: Springer International Publishing) ISBN 978-3-319-44647-9 978-3-319-44649-3 URL http://link.springer.com/10.1007/978-3-319-44649-3

[22] Chai Z, Shi B, Guo Z and Rong F 2011 *Journal of Non-Newtonian Fluid Mechanics* **166** 332–342 ISSN 03770257 URL https://linkinghub.elsevier.com/retrieve/pii/S0377025711000073
